# Simultaneous Detection of Oseltamivir- and Amantadine-Resistant Influenza by Oligonucleotide Microarray Visualization

**DOI:** 10.1371/journal.pone.0057154

**Published:** 2013-02-22

**Authors:** Yingjie Zhang, Qiqi Liu, Dou Wang, Suhong Chen, Shengqi Wang

**Affiliations:** 1 Department of Biotechnology, Beijing Institute of Radiation Medicine, Beijing, People's Republic of China; 2 Shenzhen Puruikang Biotech Co., Ltd, Shenzhen, People's Republic of China; George Mason University, United States of America

## Abstract

Presently, the resistance of Influenza A virus isolates causes great difficulty for the prevention and treatment of influenza A virus infection. It is important to establish a drug-resistance detection method for epidemiological study and personalized medicine in the clinical setting. Consequently, a cost-effective oligonucleotide microarray visualization method, which was based on quantum dot-catalyzed silver deposition, was developed and evaluated for the simultaneous detection of neuraminidase H275Y and E119V; matrix protein 2 V27A and S31N mutations of influenza A (H3N2), seasonal influenza A (H1N1), and 2009 influenza A (H1N1). Then, 307 clinical throat swab specimens were detected and the drug-resistance results showed that 100% (17/17) of influenza A (H3N2) and 100% (259/259) of 2009 influenza A (H1N1) samples were resistant to amantadine and susceptible to oseltamivir; and 100% (5/5) of seasonal influenza A (H1N1) samples were resistant to both amantadine and oseltamivir.

## Introduction

Influenza A virus significantly influences modern society and kills 500,000 to 1,000,000 people every year [1]. Though vaccination is a useful and primary strategy to control influenza pandemic, antiviral drugs have been shown to be effective for preventing and treating influenza infection. Presently, there are two categories of first-line influenza antiviral drugs used in clinical settings: neuraminidase (NA) inhibitors (oseltamivir and zanamivir) and matrix protein 2 (M2)-ion channel blockers (amantadine and rimantadine).

As these drugs are used, influenza A virus produces different proportions of drug resistance. In the USA, from 2007 to 2009, 99.4% of seasonal influenza A (H1N1) was oseltamivir resistant, and 0.4–0.7% of seasonal influenza A (H1N1) was amantadine resistant; from 2009 to 2011, 0.9–1.1% of 2009 influenza A (H1N1) (A novel swine-origin H1N1 subtype of influenza A virus emerged in Mexico in April 2009) and 0.2% of influenza A (H3N2) were oseltamivir resistant, while almost 100% of 2009 influenza A (H1N1) and influenza A (H3N2) were amantadine resistant (http://www.cdc.gov/flu/weekly/). In China, 100% of influenza A (H3N2) and 31.3–46.7% of seasonal influenza A (H1N1) was amantadine resistant in 2009 [2], [3]. None of 2009 influenza A (H1N1) and 94.4% of seasonal influenza A (H1N1) was oseltamivir resistant in 2009. Therefore, resistance detection of influenza is very important for influenza prevention, treatment, and surveillance.

Most N1 subtypes that are resistant to oseltamivir have been associated with the NA H275Y mutation [4], while N2 subtypes have been associated with the NA E119V mutation [5]. Furthermore, more than 99% of amantadine resistance generally resulted from mutations V27A and S31N in M2 [6], [7], [8]. To ensure rapid resistance diagnosis, several molecular assays based on real-time PCR, microarrays, and sequencing have been reported for the resistance detection of influenza A virus [8], [9], [10], [11]. However, to the best of our knowledge, simultaneous detection of resistance mutations of oseltamivir and amantadine on a single assay has not been reported previously.

DNA microarrays, a technology with rapid, reliable, efficient, precise, and high-throughput characteristics, have been used for gene expression analysis [12], single-nucleotide polymorphism (SNP) detection [13], disease diagnosis [14], pathogenic microorganism detection [15], etc. Quantum dots, semiconductor nanocrystal, have been widely used for diverse bio imagine applications due to their robust fluorescence characteristics [16], [17]. However, microarray visualization technology based on quantum dot-catalyzed silver deposition has rarely been reported. In this paper, this microarray visualization technology was used for drug-resistance detection of influenza A virus.

### Objective

The objective of this study was to design a cost-effective oligonucleotide microarray visualization method to simultaneously detect NA H275Y, NA E119V, M2 V27A, and M2 S31N mutations of influenza A (H3N2), seasonal influenza A (H1N1), and 2009 influenza A (H1N1).

## Materials and Methods

### Ethics Statement

This assay did not involve human participants or human experimentation. The only human materials used were throat swab samples collected from hospital patients with fever. Informed written consent was obtained from patients. Ethical approval for this research was obtained from the Research Ethics committee, Academy of Military Medical Sciences, People's Republic of China.

### Specimen collection and processing

Clinical throat swab samples of influenza A virus were collected from the Yiwu Centers for Disease Control (CDC) of Zhejiang province, People's Liberation Army 301 Hospital of China, and People's Liberation Army 307 Hospital of China from April to December 2009. Total RNA, extracted by the TIANamp Virus RNA Kit (Tiangen Biotech Beijing Co., Ltd.), was used for molecular diagnosis.

### Primer and probe design

A total of 305 NA gene and 607 M2 gene FASTA sequences of human influenza A (H3N2), seasonal influenza A (H1N1), and 2009 influenza A (H1N1) isolates were downloaded from NCBI's nucleotide database (http://www.ncbi.nlm.nih.gov/genomes/FLU/FLU.html). Then, the sequences were aligned using AlignX (a component of the Vector NTI Advance 10.3.0). We designed degenerate primers to amplify the resistance-associated sequences of oseltamivir and amantadine, respectively, in the conserved upstream and downstream regions. Moreover, microarray probes ranging from 17 to 21 nucleotides were designed to detect H275Y, E119V, V27A, and S31N mutations, respectively, of the three influenza subtypes. Eventually, nine primers and twenty-nine probes, which were able to perfectly distinguish susceptible and resistant genotypes, were selected. All the primers and probes were verified by BLAST (http://blast.ncbi.nlm.nih.gov/), and the sequences are shown in Tables 1 and 2.

**Table 1:** The primer sequences for microarray.

Primers^a^	Sequence(5′-3′)^f^	Location	Targeted genes^g^	Targeted viruses
NF1-1	CAAGAGTCTGAATGTG*CA*TG	699–718^c^	Neuraminidase	2009 influenza A (H1N1)
NF1-2	CAAGAGTCTGAATGTG*TC*TG	699–718^c^	Neuraminidase	Seasonal influenza A (H1N1)
NF1-3	CTGACCAACACCACCATA	221–238^d^	Neuraminidase	Influenza A (H3N2)
NR2-1^b^	GGATCCCAAATCATCTCAAA	1131–1150^c^	Neuraminidase	2009 influenza A (H1N1) Seasonal influenza A (H1N1)
NR2-2^b^	CATCAATAGGGTCCGATA	482–499^d^	Neuraminidase	Influenza A (H3N2)
MF2-1	CGAATGGG*G*GTGCAGATGC	752–770^e^	Matrix protein	Seasonal influenza A (H1N1) Influenza A (H3N2)
MF2-2	CGAATGGG*A*GTGCAGATGC	752–770^e^	Matrix protein	2009 influenza A (H1N1)
MR3-1^b^	TCCACAGCA*T*TCTGCTGTTCC	947–967^e^	Matrix protein	Seasonal influenza A (H1N1) Influenza A (H3N2)
MR3-2^b^	TCCACAGCA*C*TCTGCTGTTCC	947–967^e^	Matrix protein	2009 influenza A (H1N1)

a F for forward primers and R for reverse primers.

b All the reverse primers with a Cy3- or biotin-labeled 5′-end.

c Number of the position of the primer according to GenBank accession number CY081570.

d Number of the position of the primer according to GenBank accession number CY091828.

e Number of the position of the primer according to GenBank accession number HQ011421.

f Nucleotides in italic showed the natural variants of different subtypes.

g The primers for neuraminidase were used for oseltamivir-resistant mutation fragment amplification and the primers of matrix protein were used for amantadine-resistant mutation fragment amplification.

**Table 2:** The probes sequences for microarray.

Virus	Probes	Sequence(5′-3′)^a^	Location^b^
Influenza A (H3N2)	H3N2-27-W1	ACCCGCTTG**T**TGTTGC*C*	M 784-800
	H3N2-27-W2	ACCCGCTTG**T**TGTTGC*T*	M 784-800
	H3N2-27-M1	ACCCGCTTG**C**TGTTGC*C*	M 784-800
	H3N2-27-M2	ACCCGCTTG**C**TGTTGC*T*	M 784-800
	H3N2-31-W1	TTGC*C*GCGA**G**TATCATTG	M 796-813
	H3N2-31-W2	TTGC*T*GCGA**G**TATCATTG	M 796-813
	H3N2-31-M1	TTGC*C*GCGA**A**TATCATTG	M 796-813
	H3N2-31-M2	TTGC*T*GCGA**A**TATCATTG	M 796-813
	H3N2-119-W	GTGACAAGAG**A**ACCTTATGTG	NA 346-366
	H3N2-119-M	GTGACAAGAG**T**ACCTTATGT	NA 346-365
Seasonal influenza A (H1N1)	H1N1-27-W1	A*C*CCTCT*T*G**T**T*G*TTGCC	M 784-800
	H1N1-27-W2	A*T*CCTCT*T*G**T**T*G*TTGCC	M 784-800
	H1N1-27-W3	A*T*CCTCT*C*G**T**T*A*TTGCC	M 784-800
	H1N1-27-M1	A*C*CCTCT*T*G**C**T*G*TTGCC	M 784-800
	H1N1-27-M2	A*T*CCTCT*T*G**C**T*G*TTGCC	M 784-800
	H1N1-27-M3	A*T*CCTCT*C*G**C**T*A*TTGCC	M 784-800
	H1N1-31-W1	TTGCCGCAA**G**TAT*AA*TTG	M 796-813
	H1N1-31-W2	TTGCCGCAA**G**TAT*CA*TTG	M 796-813
	H1N1-31-W3	TTGCCGCAA**G**TAT*AG*TTG	M 796-813
	H1N1-31-M1	TTGCCGCAA**A**TAT*AA*TTG	M 796-813
	H1N1-31-M2	TTGCCGCAA**A**TAT*CA*TTG	M 796-813
	H1N1-275-W	ACCCAATTTT**C**ATTATGAGGA	NA 813-833
	H1N1-275-M	ACCCAATTTT**T**ATTATGAGG	NA 813-832
2009 influenza A(H1N1)	PH1N1-27-W	TCCTCTCG**T**CATTGCAG	M 785-801
	PH1N1-27-M	ATCCTCTCG**C**CATTGCA	M 784-800
	PH1N1-31-W	TGCAGCAA**G**TATCATTGG	M 797-814
	PH1N1-31-M	TTGCAGCAA**A**TATCATTGG	M 796-814
	PH1N1-275-W	CCCTAATTAT**C**ACTATGAGGA	NA 813-833
	PH1N1-275-M	CCCTAATTAT**T**ACTATGAGG	NA 813-832
Quality control^C^	20T	TTTTTTTTTT TTTTTTTTTT	

a A repeat sequence of 12T with an amino-labeled 3′-end was connected to the 3′-end of all the probes. The bold nucleotides represent the resistant or susceptible genotypes and the natural variants nucleotides were shown in italics.

b M presented Matrix protein, NA presented Neuraminidase.

C A repeat sequence of 20T with an amino-labeled 3′-end, Cy3- or Biotin-labeled 5′-end was used as microarray quality control.

### Microarray preparation

**Figure 1:**
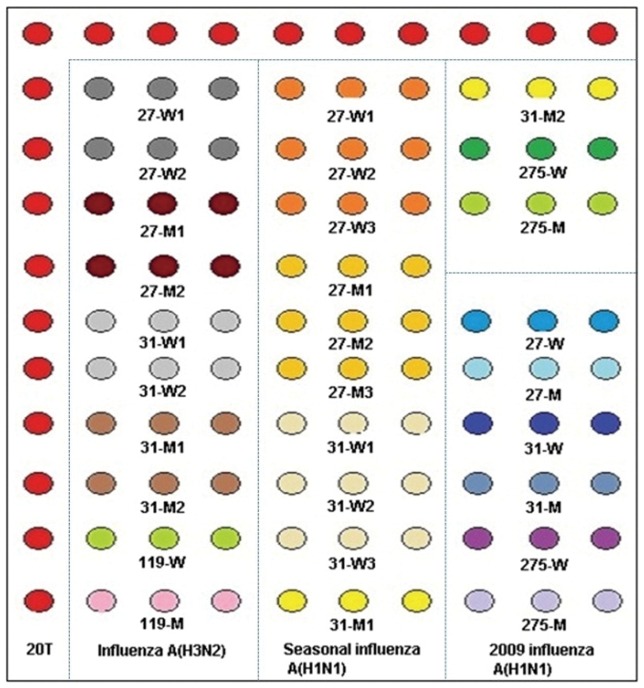
Microarray layout. Capture probes were spotted in triplicate in rows and were grouped in three areas by subtypes. The sequence of 20T was repeated 20 times for quality control and indicated the situations of capture probes.

All microarray probes were synthesized by Sangon Biotech Co., Ltd. (Shanghai), and a repeat sequence of 12T with an amino-labeled 3′-end was connected to the 3′-end of all the probes so that it could be fixed on the aldehyde-chip surface. Probes, at 50 µM final concentration, were spotted on the aldehyde-chip after mixing with uniform proportional printing buffer (5% glycerol, 0.1% sodium dodecyl sulfate (SDS), 6×saline-sodium citrate buffer (SSC), and 2% (wt/vol) Ficoll 400). The microarray was placed in a dryer for 24 h at room temperature, and unbound probes were removed by washing once with 0.2% SDS and once with distilled water for 30 s each at room temperature prior to use. The microarray layout is shown in Fig. 1.

### RT-PCR amplification

The resistance mutation fragments of oseltamivir and amantadine were respectively amplified by a RT-PCR system. Each RT-PCR was performed in a 20 µL reaction volume containing 10 µL of 2×1 Step Buffer, 5 µL of genomic RNA template, 0.8 µL of PrimeScript 1 Step Enzyme Mix (Takara Biotechnology (Dalian) Co., Ltd.). For the oseltamivir-resistant mutation fragment amplification, the RT-PCR system contained each reverse primer at 0.5 µM and each forward primer at 0.1 µM; while for the amantadine-resistant mutation fragment amplification, it contained each reverse primer at 0.75 µM and each forward primer at 0.15 µM. Amplifications were performed on a Veritil 96-Well Thermal Cycler PCR system (Applied Biosystems) using the following conditions: 30 min at 50°C; 2 min at 94°C; 45 cycles of 20 s at 94°C, 20 s at 55°C, and 20 s at 72°C; and a final extension of 5 min at 72°C.

### Hybridization and signal detection

After the resistance mutation fragments were amplified, 2.5 µL of each amplification product of the two reactions was mixed with 5 µL of hybridization buffer (8×SSC, 0.6% SDS, 10% formylamine, and 10×Denhardt). A total of 10 µL of hybridization mixture was added to the hybridization region on the microarray, then the chip was placed in the hybrid-box, and it was incubated for 1 h at 45°C. Subsequently, the chip was washed once in turn for 30 s with 1×SSC and 0.2% SDS, 0.2×SSC, and 0.1×SSC at room temperature.

In this assay, we introduced two approaches (fluorescence and visible detection) to detect the microarray signal. For the fluorescence method, Cy3-labeled reverse primers were used in the RT-PCR amplification, and after hybridization and washing, the dried chip was directly scanned by a GenePix Personal 4100A (Axon Instrument). For the visible detection method, biotin-labeled reverse primers were used in the RT-PCR amplification. After hybridization and washing were complete, the chip was incubated with 15 µL of 25 nM streptavidin-quantum dots (Str-QDs, Wuhan Jiayuan Quantum Dots Co., Ltd.) for 10 min at 37°C. Then, the chip was washed with PBST (phosphate buffer, 0.05% Tween 20) five times for 20 s and distilled water once for 10 s at room temperature. Subsequently, 15 µL of aqueous silver acetate solution (Acros Organics) and 15 µL of hydroquinone citric acid solution (Acros Organics) were mixed before use and added. Eventually, the chip was washed with distilled water to terminate the reaction when the black signal point appeared. The dried chip was scanned by Image Scanner (UMAX, Amersham Biosciences).

The probe signal densities of the two microarray detection methods were calculated by Arrayvision 7.0.

### Specificity and sensitivity evaluation

The specificity of this microarray was evaluated by positive strains of influenza (see in Table 3) and a panel of negative controls. These negative controls include common human respiratory viruses such as influenza B, parainfluenza 1, 2, 3, adenovirus AD2, AD3, AD30, AD40, AD41, measles, rubella, parotitis, respiratory syncytial virus HK6 and B. *In vitro* transcribed RNAs of oseltamivir and amantadine-susceptible and resistant genotypes, which were defined by sequencing, were also used as templates to determine the reliability of genotypes detection results.

**Table 3:** Positive strains of influenza A virus.

Subtypes	Strains^a^
2009 influenza A (H1N1)	A/Beijing/SWL1/2009/(H1N1)
	A/Hunan/SWL3/2009/(H1N1)
seasonal influenza A (H1N1)	A/Hufang/7/1999(H1N1)
influenza A (H3N2)	A/Yunnan/1145/2005/(H3N2)

a The positive strains of influenza A virus were collected to develop and evaluate the microarray.

In these assays, Influenza A virus (H1N1) Nucleic Acid Detection Kit (coProbes Real-Time PCR) (Shenzhen Puruikang Biotech Co., Ltd.) and Influenza virus A Real-Time RT-PCR Kit (Shanghai ZJ Bio-Tech Co., Ltd.) were used as references for sensitivity evaluation. In particular, five dilutions (initial, 5^1^, 5^2^, 5^3^, and 5^4^) of the RNA templates, the clinical throat swab sample extracts of the three influenza A virus subtypes, were amplified to compare the sensitivity of our microarray with that of the Real-Time RT-PCR Kit. Subsequently, some of the samples detected by microarray analysis were sequenced to verify the subtypes.

*In vitro* transcribed RNAs (10^6^ copies/µl) of oseltamivir and amantadine-susceptible and resistant genotypes mixed at different proportions (99∶1, 95∶5, 90∶10, 50∶50, 10∶90, 5∶95, 1∶99) were used as templates to evaluate the sensitivity of the microarray to distinguish the mixed population.

### 3.8 Resistance detection of clinical samples

The resistance genotypes of 307 clinical throat swab samples of influenza A virus, collected from three institutions, were detected by microarray analysis, and the resistance genotypes of a portion of samples were verified by sequencing.

## Results

### Specificity of the microarray

**Figure 2:**
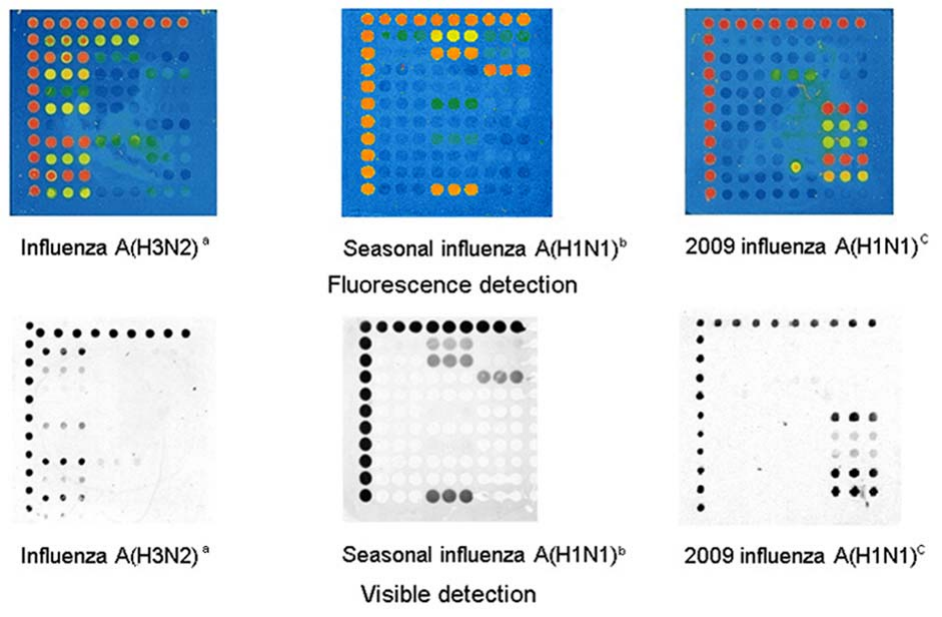
Fluorescence and visible detection of three subtypes of influenza A virus. ^a b c^ All of the three viruses used for comparing the accuracy between fluorescence and visible detection were clinical throat swab samples.

In this assay, the microarray was able to well distinguish the subtypes and resistance genotypes of influenza A (H3N2), seasonal influenza A (H1N1), and 2009 influenza A (H1N1) samples. The ratio of the arithmetic mean of all the wild probes to that of all the mutant probes for the definite subtypes was determined. If the ratio was greater than 2.0, the sample was considered to be wild-type, while if the ratio was less than 0.5, the sample was considered to be resistant. The microarray images of the three subtypes are shown in Fig. 2.

The results of susceptible and resistant subtypes for *in vitro* transcribed RNAs showed the microarray was able to exactly distinguish the variants of these nucleotides (see in Fig. S1). All the negative controls showed the negative microarray results and which also demonstrated the specificity of this assay (see in Fig. S2).

### Sensitivity of the microarray

**Figure 3:**
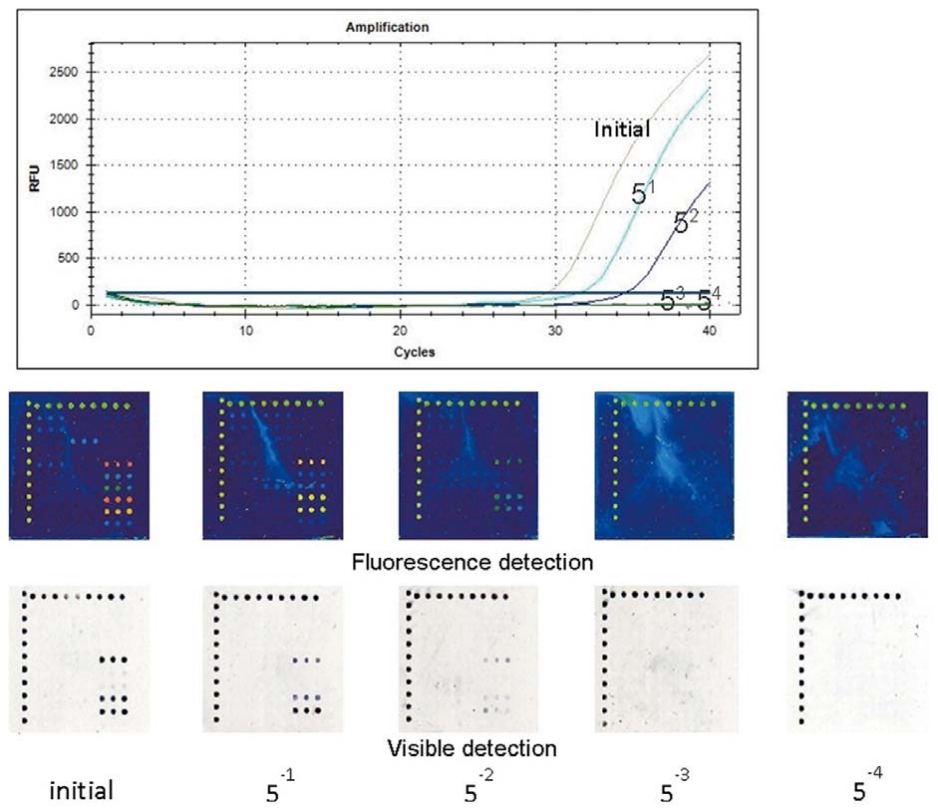
The sensitivity comparison results of 2009 influenza A (H1N1)^a^. ^a^ The real-time RT-PCRs were amplified by the CFX96 Touch™ Real-Time PCR Detection System (Bio-Rad). Five dilutions (initial, 5^1^, 5^2^, 5^3^, and 5^4^) of 2009 influenza A (H1N1) templates, the extracts of clinical throat swab samples, were amplified to compare the sensitivity of our microarray with that of the Influenza A virus (H1N1) Nucleic Acids Detection Kit (coProbes Real-Time PCR) (Shenzhen Puruikang Biotech Co., Ltd.). All three methods could detect templates at 5^2^ dilutions, so they had similar sensitivities. The sensitivity comparison results of the other two subtypes showed similar conclusions (the results were no shown).

For the sensitivity determination, we compared the two types of microarray detection methods with the real-time RT-PCR method, and we discovered that the Str-QDs and Cy3 methods possessed similar detection sensitivities as the real-time RT-PCR method. The sensitivity of the real-time RT-PCR kit was 1.0×10^3^ PFU/mL; consequently, our microarrays had similar sensitivities. The sensitivity comparison results of 2009 influenza A (H1N1) subtypes are shown in Fig. 3.

The sensitivity of the method to distinguish the mixed population of the drug-sensitive and resistant was compared by detection of the mixed *in vitro* transcribed RNAs templates, and the results showed that the microarray detected the minor population (>1% for oseltamivir mixed RNA and >5% for amantadine mixed RNA; the compare results of 2009 influenza A (H1N1) see in Fig. S3).

### Resistance detection of clinical samples

**Figure 4:**
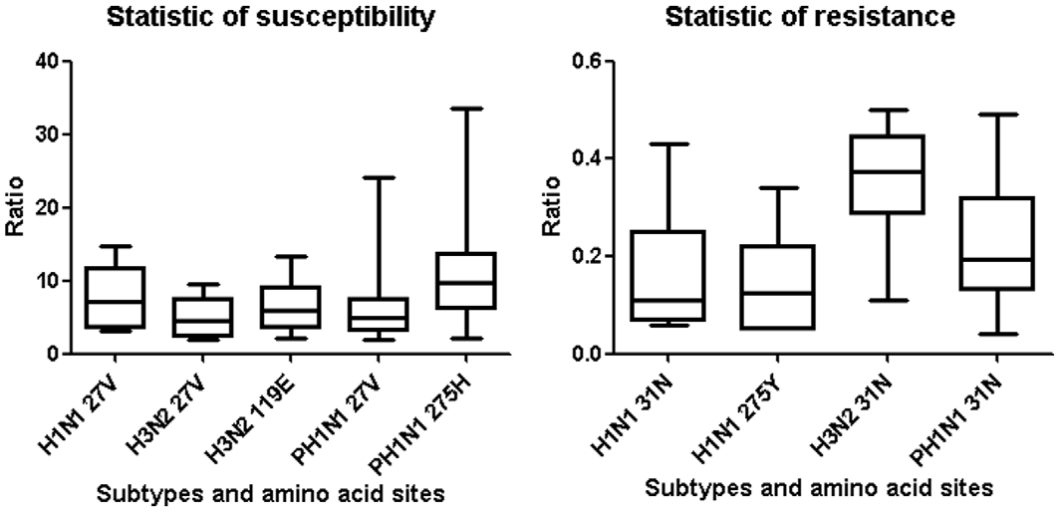
The ratio statistic results of resistance and susceptibility of the clinical throat swap specimens. The ratio of the arithmetic mean of all the wild probes to that of all the mutant probes for the definite subtypes was determined and the statistic results of 281 positive clinical throat swab samples were separately shown in two column graphs by the ratio range of resistance and susceptibility. The statistics showed that the ratios of wild-type sites were greater than 2 and these of mutant-type sites were less than 0.5.

A total of clinical throat swab specimens of three subtypes of influenza A virus, collected from the hospital patients with fever who couldn't determine whether taking medication, were analyzed by our microarray. The subtypes results of these samples were 17 influenza A (H3N2), 5 seasonal influenza A (H1N1), 259 2009 influenza A (H1N1), and 26 negative and the drug-resistance results were as follows: 100% (17/17) influenza A (H3N2), 100% (5/5) seasonal influenza A (H1N1), and 100% (259/259) 2009 influenza A (H1N1) clinical throat swab samples were resistant to amantadine; 100% (5/5) seasonal influenza A (H1N1) samples were resistant to oseltamivir; and no influenza A (H3N2) or 2009 influenza A (H1N1) samples were resistant to oseltamivir. The resistance genotypes of 161 positive samples of the three subtypes were verified by sequencing (see in Fig. S4). V27A sites of two cases of 2009 influenza A (H1N1) (27V confirmed by sequencing) were not detected by microarray, while 31N and 275H were consistent with sequencing. The accuracy of consistency between sequencing and microarray was 98.8%. There were three genotypes of the 281 positive clinical throat swab samples: 27V-31N-119E for influenza A (H3N2), 27V-31N-275Y for seasonal influenza A (H1N1), and 27V-31N-275H for 2009 influenza A (H1N1) and all the statistics results of ratio for clinical throat swab samples are shown in column graphs (see in Fig. 4). The statistics showed that the ratios of wild-type sites were greater than 2 and these of mutant-type sites were less than 0.5.

All of the cycle threshold (Ct) values of the 26 negative samples, detected by real-time RT-PCR, were more than 35. The microarray detection results of clinical samples are shown in Table 4.

**Table 4:** The subtypes and resistant genotypes results of clinical throat swab samples.

	Microarray			Sequencing
Subtypes	Number	Amantadine-resistance(resistance rates)	Oseltamivir-resistance(resistance rates)	Number(Coincidence rates)
Influenza A (H3N2)	17	17(100%)	0(0%)	14(100%)
Seasonal influenza A (H1N1)	5	5(100%)	5(100%)	3(100%)
2009 influenza A (H1N1)	259	259(100%)	0(0%)	144(98.6%)
negative	26	-	-	
total	307	281	5	161(98.8%)

All the positive samples were resistant to amantadine, none of influenza A (H3N2) and 2009 influenza A (H1N1) were resistant to oseltamivir, but all the five seasonal influenza A (H1N1), collected in 2009, were resistant to both oseltamivir and amantadine. V27A sites of two cases of 2009 influenza A (H1N1) (27V confirmed by sequencing) were not detected by microarray, while 31N and 275H were consistent with sequencing. The accuracy of consistency between sequencing and microarray was 98.8%.

## Discussion

To the best of our knowledge, this is the first report of a method that simultaneously detects two types of influenza antiviral drug-resistance mutations of three influenza subtypes on a single DNA microarray. This microarray was fast and high-throughput, and the entire experiment, from extraction of samples to microarray detection, could be completed within 6 h. The detection cost per sample was less than five dollars. These characteristics will aid the treatment, prevention, resistance surveillance and epidemiological study of influenza A virus. In order to determine the reliability of the microarray, *in vitro* transcribed RNAs of oseltamivir and amantadine-susceptible and resistant genotypes, which were defined by sequencing, were used as templates to verify the detection results. Some positive strains of influenza A virus were also collected to verify the microarray results. Moreover, the sequencing results of 161 positive samples also verified the reliability of the microarray results and the accuracy between sequencing and microarray was 98.8%. But this accuracy result has some limitation due to the scant date of influenza A virus (H3N2) and influenza A virus (H1N1). In this assay, we compared the sensitivity and genotyping of two kinds of microarray detection methods, and they had similar sensitivities as the real-time RT-PCR kit. Though the Cy3 method had a shorter detection time and the simpler detection step, it required an expensive fluorescence scanner. In contrast, the Str-QDs approach could significantly reduce testing costs, and it also could be used for field diagnostics because the results can be visualized by the naked eye. Therefore, funded institutions are able to choose the Cy3 method, while other users can choose the lower cost Str-QDs method.

In this assay, multiple probe pairs were used for amantadine resistance detection of influenza A (H3N2) and seasonal influenza A (H1N1) since the existence of natural variants nucleotides near the resistance mutations and that maybe a limitation of DNA microarray methods compare with sequencing methods. However, for the mixed infection samples (infected two or more influenza A virus at the same time), which could not be detected by direct sequencing of PCR products, were able to well distinguish the subtypes and resistance genotypes by microarray. All of the cycle threshold (Ct) values of the 26 negative samples, detected by real-time RT-PCR, were more than 35, so the negative results detected by microarray may be due to these reasons: the concentrations of these samples below the microarray sensitivity, infected with other subtypes of influenza virus, or indeed negative samples. In addition, a small number of high viral load specimens, which could not exactly distinguish the resistance genotypes, could be diluted 20-fold before or after RT-PCR amplification to increase the discriminatory power.

NA inhibitors and M2-ion channel blockers are two classes of antiviral drugs that have been approved for specific management of influenza. In this study, all of the clinical throat swab samples were resistant to amantadine and none of the influenza A (H3N2) and 2009 influenza A (H1N1) samples were resistant to oseltamivir. However, all of the five seasonal influenza A (H1N1) samples, collected in 2009, were resistant to both oseltamivir and amantadine. Thus, zanamivir or other drugs should be selected to treat these dual-resistant virus-infected patients. However, the date for seasonal A (H1N1) was scant because only five seasonal A (H1N1) viruses were tested. Since 2009, influenza A (H3N2) and 2009 influenza A (H1N1) have increased more rapidly than seasonal influenza A (H1N1) to become the predominant epidemic strains of influenza A virus. According to the literature, multiple amino acid mutations are associated with drug-resistance of influenza A virus. In addition to the resistance mutations detected in this paper, there are some rare mutations that could lead to drug-resistance. For instance, I117V [18], I117M [18], S247N [19], I223R [20], N294S [21], and R292K [22] of NA have been reported to be associated with NA inhibitor resistance, and some of them had combinatorial, compensatory, or synergistic effects [18], [23], [24]. These effects significantly increased the virulence or resistance of influenza virus. Furthermore, the mutations associated to the response of influenza A (H5N1) and Influenza B virus to anti-viral drugs also could not be detected by this assay, which limited the suitable extent of the microarray. In this paper, oseltamivir-resistant clinical samples of influenza A (H3N2) and 2009 influenza A (H1N1) were not detected, but due to the limited number of specimens, the short time frame of samples collection, and the limited detection sites associate to resistance, we were not able to demonstrate any oseltamivir-resistant strains in China.

Presently, NA inhibitors are still considered to be the most effective drugs in treating and preventing infection of influenza A (H3N2) and 2009 influenza A (H1N1); and although M2-ion channel blockers have a high proportion of resistance, they are still commonly used in clinical settings in China. According to report, susceptible and resistant influenza infection studies in mouse models have shown that the efficacy of reducing mortality and weight loss with a combination of amantadine with oseltamivir and ribavirin was significantly higher than that of dual and single drug treatment; in addition, they demonstrated that the activity of amantadine against a resistant strain could be restored with the triple combination [25]. Furthermore, the combination of the three antiviral drugs could enhance a high genetic barrier to resistance; consequently, it continually suppressed drug-resistant viruses [26]. A clinical retrospective report also showed that the 14-day mortality of patients who received the triple-combination of the three antiviral drugs was significantly lower than that of patients who received oseltamivir mono-therapy [27]. Thus, although amantadine has a very high rate of resistance, it still possesses a great significance in the clinical treatment of influenza A virus infection.

For immune-deficient and severe hospitalized patients, rapid determination of virus resistance and monitoring viral clearance are extremely important. Furthermore, since currently there exists a shortage of antiviral drugs, resistance surveillance is essential for establishing appropriate treatment plans for patients, standardizing the application of antiviral drugs, and preventing abuse. Presently, hundreds of oseltamivir-resistant cases have been reported throughout the world [28], [29], [30]; so if we do not regulate the use of oseltamivir, the same situation as with amantadine could result in the near future – all influenza viruses could be resistant to oseltamivir.

## Supporting Information

Figure S1 **The microarray and sequencing results to detect susceptible and resistant templates of** ***in vitro*** **transcribed RNAs.** *In vitro* transcribed RNAs of oseltamivir and amantadine-susceptible and resistant genotypes, which were defined by sequencing, were used as templates to determine the reliability of genotypes detection results. The results showed that the microarray was able to exactly distinguish the variants of these susceptible and resistant templates.
(PDF)

Figure S2 **The microarray results of a panel of negative controls.** The specificity of this microarray was evaluated by a panel of negative controls, which include common human respiratory viruses such as influenza B, parainfluenza 1, 2, 3, adenovirus AD2, AD3, AD30, AD40, AD41, measles, rubella, parotitis, respiratory syncytial virus HK6 and B. The microarray results of these negative controls demonstrated the specificity of this assay.
(PDF)

Figure S3 **The sensitivity of the method to distinguish the mixed population of 2009 influenza A (H1N1).** *In vitro* transcribed RNAs (10^6^ copies/µl) of oseltamivir and amantadine-susceptible and resistant genotypes mixed at different proportions (99∶1, 95∶5, 90∶10, 50∶50, 10∶90, 5∶95, 1∶99) were used as templates to evaluate the sensitivity of the microarray to distinguish the mixed population. The results showed that the microarray detected the minor population (>1% for oseltamivir mixed RNA and >5% for amantadine mixed RNA).
(PDF)

Figure S4 **The sequencing results of 161 positive samples of influenza A virus.** 144 2009 influenza A (H1N1), 14 influenza A (H3N2), and 3 Seasonal influenza A (H1N1) positive clinical throat swab samples were verified by sequencing using BigDye terminator cycle sequencing kit, version 3.1 (Applied Biosystems) in ABI 3730 Genetic analyzer (Applied Biosystems), then the sequences were aligned by AlignX (a component of Vector NTI Advance 10.3.0) respectively.
(PDF)
