# Supplementary material for: Simultaneous Detection of Oseltamivir- and Amantadine-Resistant Influenza by Oligonucleotide Microarray Visualization
Source: PLoS One. 2013 Feb 22;8(2):e57154. doi: 10.1371/journal.pone.0057154 (PMC3579783; doi:10.1371/journal.pone.0057154)
Supplement: Figure S1 — The microarray and sequencing results to detect susceptible and resistant templates of in vitro transcribed RNAs. In vitro transcribed RNAs of oseltamivir and amantadine-susceptible and resistant genotypes, which were defined by sequencing, were used as templates to determine the reliability of genotypes detection results. The results showed that the microarray was able to exactly distinguish the variants of these susceptible and resistant templates. (PDF) [file pone.0057154.s001.pdf]

The microarray and sequencing results to detect susceptible and resistant templates of *in vitro* transcribed RNAs

The microarray results of *in vitro* transcribed RNAs

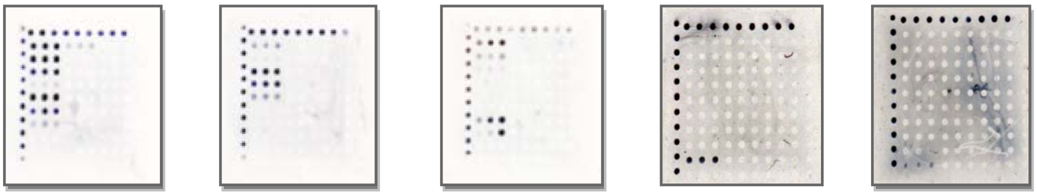

H3N2-27V-31S | H3N2-27A-31S | H3N2-27V-31N | H3N2-119E | H3N2-119V

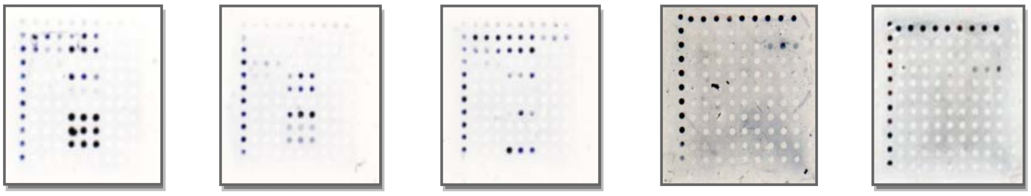

H1N1-27V-31S | H1N1-27A-31S | H1N1-27V-31N | H1N1-274H | H1N1-274Y

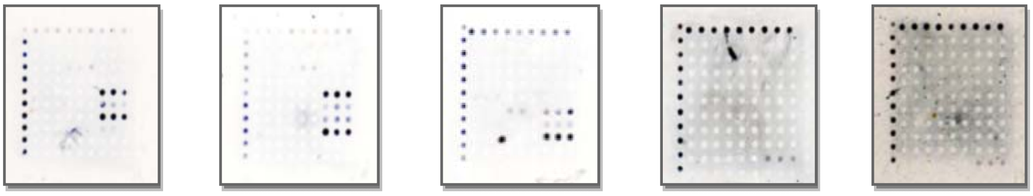

PH1N1-27V-31S | PH1N1-27V-31N | PH1N1-27A-31N | PH1N1-274H | PH1N1-274Y

The sequence of plasmid templates used for *in vitro* transcription.

Influenza A(H3N2)

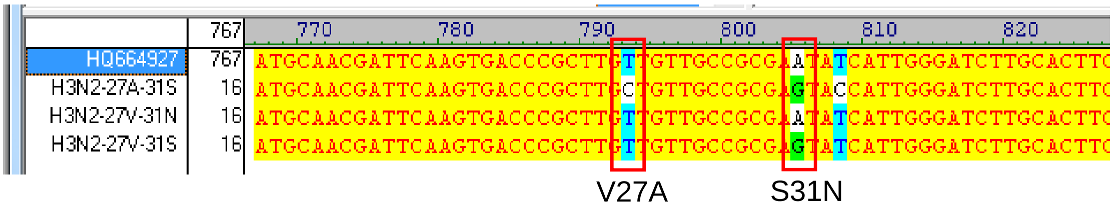

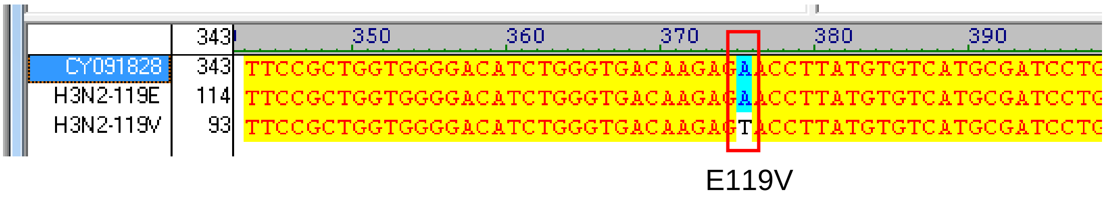

Seasonal influenza A(H1N1)

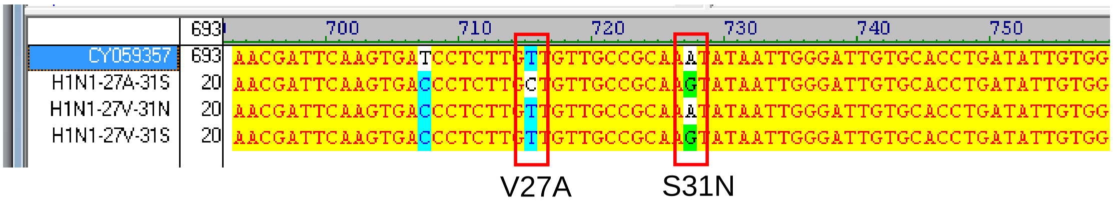

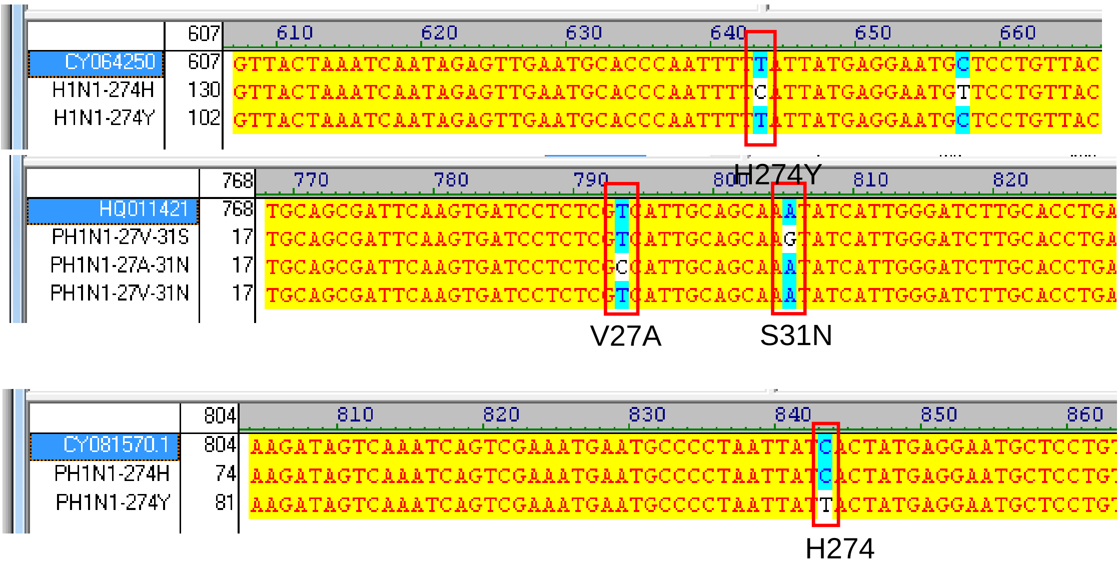

607 610 620 630 640 650 660
CY064250 607 GTTACTAAATCAATAGAGTTGAATGCACCCAATTTTTATTATGAGGAATGCTCCTGTTAC
H1N1-274H 130 GTTACTAAATCAATAGAGTTGAATGCACCCAATTTTCATTATGAGGAATGTTCCTGTTAC
H1N1-274Y 102 GTTACTAAATCAATAGAGTTGAATGCACCCAATTTTTATTATGAGGAATGCTCCTGTTAC
H274Y
768 770 780 790 800 810 820
HQ011421 768 TGCAGCGATTCAAGTGATCCTCTCGTGATTGCAGCAAATATCATTGGGATCTTGCACCTGA
PH1N1-27V-31S 17 TGCAGCGATTCAAGTGATCCTCTCGTGATTGCAGCAAGTATCATTGGGATCTTGCACCTGA
PH1N1-27A-31N 17 TGCAGCGATTCAAGTGATCCTCTCGCGATTGCAGCAAATATCATTGGGATCTTGCACCTGA
PH1N1-27V-31N 17 TGCAGCGATTCAAGTGATCCTCTCGTGATTGCAGCAAATATCATTGGGATCTTGCACCTGA
V27A
S31N
804 810 820 830 840 850 860
CY081570.1 804 AAGATAGTCAAATCAGTCGAAATGAATGCCCCTAATTATCACTATGAGGAATGCTCCTG
PH1N1-274H 74 AAGATAGTCAAATCAGTCGAAATGAATGCCCCTAATTATCACTATGAGGAATGCTCCTG
PH1N1-274Y 81 AAGATAGTCAAATCAGTCGAAATGAATGCCCCTAATTATTACTATGAGGAATGCTCCTG
H274
