# Supplementary figures and images for: Simultaneous Detection of Oseltamivir- and Amantadine-Resistant Influenza by Oligonucleotide Microarray Visualization
Source: PLoS One. 2013 Feb 22;8(2):e57154. doi: 10.1371/journal.pone.0057154 (PMC3579783; doi:10.1371/journal.pone.0057154)

The microarray results of a panel of negative controls

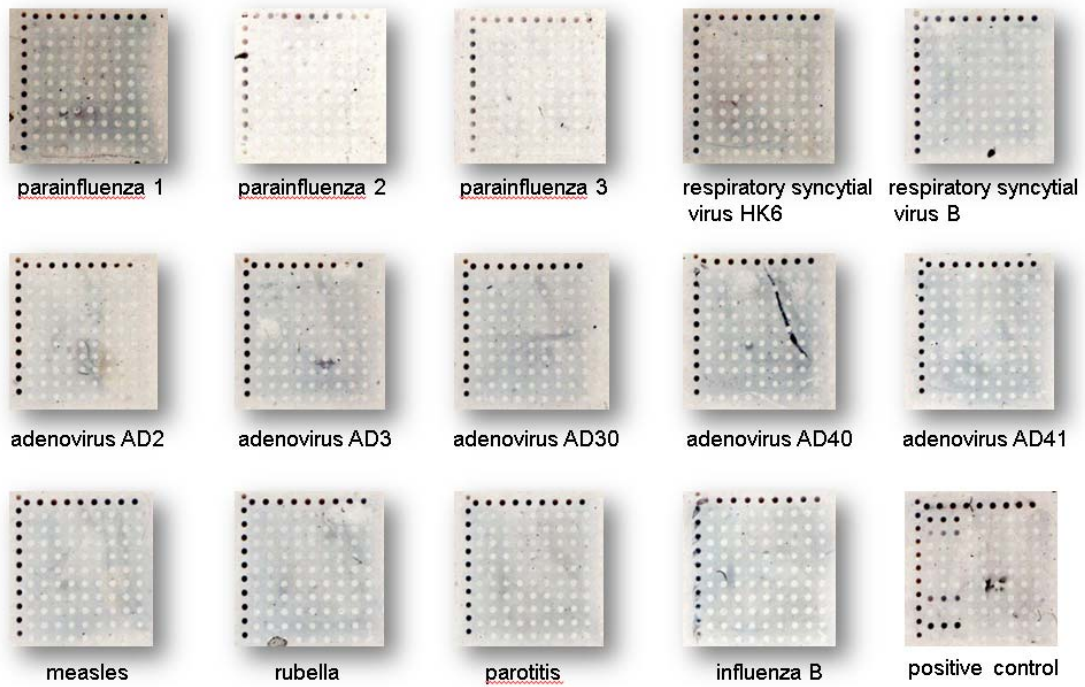

Supplement: Figure S2 — The microarray results of a panel of negative controls. The specificity of this microarray was evaluated by a panel of negative controls, which include common human respiratory viruses such as influenza B, parainfluenza 1, 2, 3, adenovirus AD2, AD3, AD30, AD40, AD41, measles, rubella, parotitis, respiratory syncytial virus HK6 and B. The microarray results of these negative controls demonstrated the specificity of this assay. (PDF) [file pone.0057154.s002.pdf]
