# Supplementary material for: Simultaneous Detection of Oseltamivir- and Amantadine-Resistant Influenza by Oligonucleotide Microarray Visualization
Source: PLoS One. 2013 Feb 22;8(2):e57154. doi: 10.1371/journal.pone.0057154 (PMC3579783; doi:10.1371/journal.pone.0057154)
Supplement: Figure S3 — The sensitivity of the method to distinguish the mixed population of 2009 influenza A (H1N1). In vitro transcribed RNAs (106 copies/µl) of oseltamivir and amantadine-susceptible and resistant genotypes mixed at different proportions (99∶1, 95∶5, 90∶10, 50∶50, 10∶90, 5∶95, 1∶99) were used as templates to evaluate the sensitivity of the microarray to distinguish the mixed population. The results showed that the microarray detected the minor population (>1% for oseltamivir mixed RNA and >5% for amantadine mixed RNA). (PDF) [file pone.0057154.s003.pdf]

The sensitivity of the method to distinguish the mixed population of 2009 influenza A (H1N1)

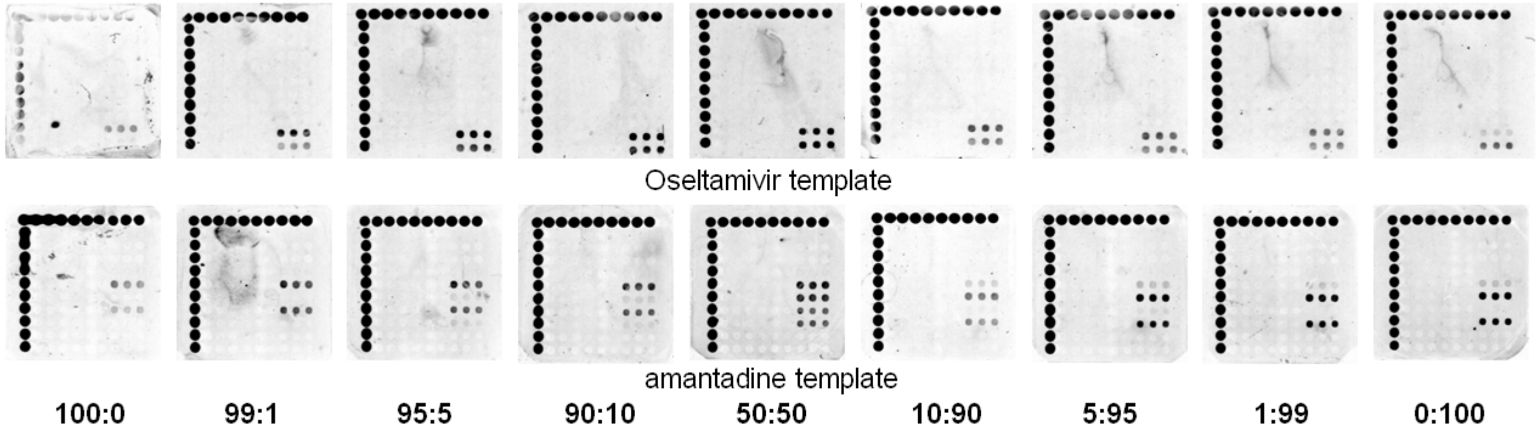

The results showed that the microarray detected the minor population in mixed template when it was more than 1% for oseltamivir (top line) and 5% for amantadine (underside line).
