# Supplementary material for: Simultaneous Detection of Oseltamivir- and Amantadine-Resistant Influenza by Oligonucleotide Microarray Visualization
Source: PLoS One. 2013 Feb 22;8(2):e57154. doi: 10.1371/journal.pone.0057154 (PMC3579783; doi:10.1371/journal.pone.0057154)
Supplement: Figure S4 — The sequencing results of 161 positive samples of influenza A virus. 144 2009 influenza A (H1N1), 14 influenza A (H3N2), and 3 Seasonal influenza A (H1N1) positive clinical throat swab samples were verified by sequencing using BigDye terminator cycle sequencing kit, version 3.1 (Applied Biosystems) in ABI 3730 Genetic analyzer (Applied Biosystems), then the sequences were aligned by AlignX (a component of Vector NTI Advance 10.3.0) respectively. (PDF) [file pone.0057154.s004.pdf]

The sequencing results of 161 positive samples of influenza A virus.

Influenza A (H3N2) M gene

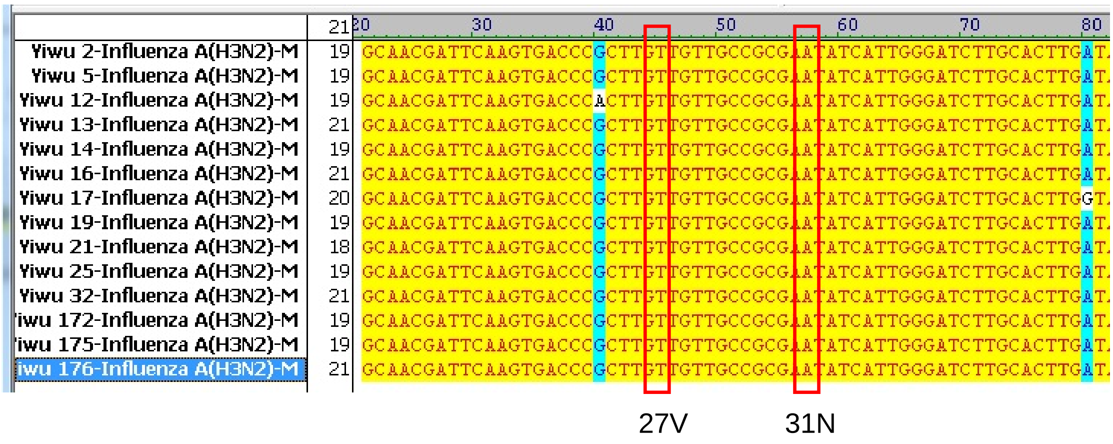

Influenza A (H3N2) NA gene

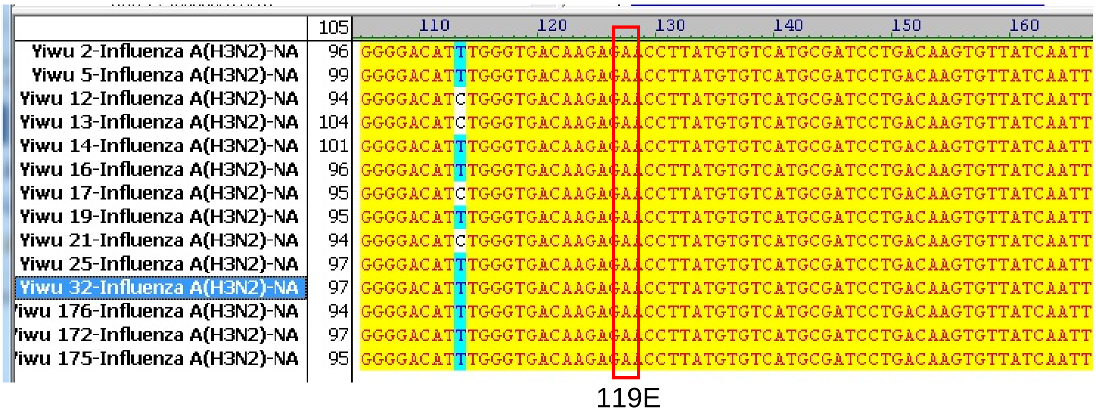

Seasonal influenza A (H1N1) M gene

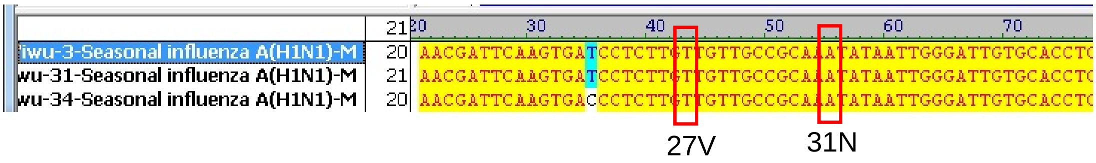

Seasonal influenza A (H1N1) NA gene

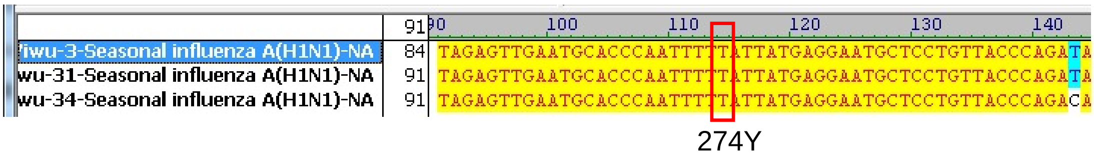

2009 influenza A (H1N1) M gene

	(41)	41 50 60 70 80
301-75-2009 influenza A(H1N1)-M	(38)	TCTCGTCATTGCAGCAAATATCATTGGGATCTTGCMCCTG
301-2654-2009 influenza A(H1N1)-M	(36)	TCTCGTCATTGCAGCAAATATCATTGGGATCTTGCACCTG
301-2655-2009 influenza A(H1N1)-M	(36)	TCTCGTCATTGCAGCAAATATCATTGGGATCTTGCACCTG
301-2747-2009 influenza A(H1N1)-M	(38)	TCTCGTCATTGCAGCAAATATCATTGGGATCTTGCACCTG
301-2749-2009 influenza A(H1N1)-M	(38)	TCTCGTCATTGCAGCAAATATCATTGGGATCTTGCACCTG
301-2750-2009 influenza A(H1N1)-M	(38)	TCTCGTCATTGCAGCAAATATCATTGGGATCTTGCACCTG
301-2751-2009 influenza A(H1N1)-M	(38)	TCTCGTCATTGCAGCAAATATCATTGGGATCTTGCACCTG
301-2760-2009 influenza A(H1N1)-M	(38)	TCTCGTCATTGCAGCAAATATCATTGGGATCTTGCACCTG
301-2767-2009 influenza A(H1N1)-M	(38)	TCTCGTCATTGCAGCAAATATCATTGGGATCTTGCACCTG
301-2770-2009 influenza A(H1N1)-M	(38)	TCTCGTCATTGCAGCAAATATCATTGGGATCTTGCACCTG
301-2777-2009 influenza A(H1N1)-M	(38)	TCTCGTCATTGCAGCAAATATCATTGGGATCTTGCACCTG
301-2796-2009 influenza A(H1N1)-M	(38)	TSTCGTCATTGCAGCAAATATCATTGGGATCTTGCACCTG
301-2807-2009 influenza A(H1N1)-M	(38)	TCTCGTCATTGCAGCAAATATCATTGGGATCTTGCACCTG
301-2833-2009 influenza A(H1N1)-M	(38)	TCTCGTCATTGCAGCAAATATCATTGGGATCTTGCACCTG
301-2835-2009 influenza A(H1N1)-M	(38)	TCTCGTCATTGCAGCAAATATCATTGGGATCTTGCACCTG
301-2836-2009 influenza A(H1N1)-M	(38)	TCTCGTCATTGCAGCAAATATCATTGGGATCTTGCACCTG
301-2837-2009 influenza A(H1N1)-M	(38)	TCTCGTCATTGCAGCAAATATCATTGGGATCTTGCACCTG
301-2838-2009 influenza A(H1N1)-M	(38)	TCTCGTCATTGCAGCAAATATCATTGGGATCTTGCACCTG
301-2840-2009 influenza A(H1N1)-M	(38)	TCTCGTCATTGCAGCAAATATCATTGGGATCTTGCACCTG
301-2856-2009 influenza A(H1N1)-M	(38)	TCTCGTCATTGCAGCAAATATCATTGGGATCTTGCACCTG
301-2858-2009 influenza A(H1N1)-M	(38)	TCTCGTCATTGCAGCAAATATCATTGGGATCTTGCMCCTG
301-2860-2009 influenza A(H1N1)-M	(38)	TCTCGTCATTGCAGCAAATATCATTGGGATCTTGCACCTG
301-2867-2009 influenza A(H1N1)-M	(38)	TCTCGTCATTGCAGCAAATATCATTGGGATCTTGCACCTG
301-2884-2009 influenza A(H1N1)-M	(38)	TCTCGTCATTGCAGCAAATATCATTGGGATCTTGCACCTG
301-2886-2009 influenza A(H1N1)-M	(38)	TCTCGTCATTGCAGCAAATATCATTGGGATCTTGCACCTG
301-2887-2009 influenza A(H1N1)-M	(38)	TCTCGTCATTGCAGCAAATATCATTGGGATCTTGCACCTG
301-2930-2009 influenza A(H1N1)-M	(40)	TCTCGTCATTGCAGCAAATATCATTGGGATCTTGCACCTG
301-2931-2009 influenza A(H1N1)-M	(38)	TCTCGTCATTGCAGCAAATATCATTGGGATCTTGCACCTG
301-2932-2009 influenza A(H1N1)-M	(38)	TCTCGTCATTGCAGCAAATATCATTGGGATCTTGCACCTG
301-3291-2009 influenza A(H1N1)-M	(38)	TCTCGTCATTGCAGCAAATATCATTGGGATCTTGCACCTG
301-3294-2009 influenza A(H1N1)-M	(38)	TCTCGTCATTGCAGCAAATATCATTGGGATCTTGCACCTG
301-3310-2009 influenza A(H1N1)-M	(38)	TCTCGTCATTGCAGCAAATATCATTGGGATCTTGCACCTG
301-3320-2009 influenza A(H1N1)-M	(38)	TCTCGTCATTGCAGCAAATATCATTGGGATCTTGCACCTG
301-3322-2009 influenza A(H1N1)-M	(38)	TCTCGTCATTGCAGCAAATATCATTGGGATCTTGCACCTG
301-3324-2009 influenza A(H1N1)-M	(38)	TCTCGTCATTGCAGCAAATATCATTGGGATCTTGCACCTG
301-3327-2009 influenza A(H1N1)-M	(38)	TCTCGTCATTGCAGCAAATATCATTGGGATCTTGCACCTG
301-3332-2009 influenza A(H1N1)-M	(38)	TCTCGTCATTGCAGCAAATATCATTGGGATCTTGCACCTG
301-3336-2009 influenza A(H1N1)-M	(38)	TCTCGTCATTGCAGCAAATATCATTGGGATCTTGCACCTG
301-3337-2009 influenza A(H1N1)-M	(38)	TCTCGTCATTGCAGCAAATATCATTGGGATCTTGCACCTG
301-6078-2009 influenza A(H1N1)-M	(38)	TCTCGTCATTGCAGCAAATATCATTGGGATCTTGCACCTG
301-6202-2009 influenza A(H1N1)-M	(38)	TCTCGTCATTGCAGCAAATATCATTGGGATCTTGCACCTG
301-6223-2009 influenza A(H1N1)-M	(39)	TCTCGTCATTGCAGCAAATATCATTGGGATCTTGCACCTG
301-6228-2009 influenza A(H1N1)-M	(38)	TCTCGTCATTGCAGCAAATATCATTGGGATCTTGCACCTG
301-6241-2009 influenza A(H1N1)-M	(38)	TCTCGTCATTGCAGCAAATATCATTGGGATCTTGCACCTG
301-6259-2009 influenza A(H1N1)-M	(38)	TCTCGTCATTGCAGCAAATATCATTGGGATCTTGCACCTG
301-6284-2009 influenza A(H1N1)-M	(38)	TCTCGTCATTGCAGCAAATATCATTGGGATCTTGCACCTG
301-6298-2009 influenza A(H1N1)-M	(38)	TCTCGTCATTGCAGCAAATATCATTGGGATCTTGCACCTG
301-6319-2009 influenza A(H1N1)-M	(38)	TCTCGTCATTGCAGCAAATATCATTGGGATCTTGCACCTG
301-6336-2009 influenza A(H1N1)-M	(38)	TCTCGTCATTGCAGCAAATATCATTGGGATCTTGCACCTG
301-6379-2009 influenza A(H1N1)-M	(38)	TCTCGTCATTGCAGCAAATATCATTGGGATCTTGCACCTG
301-6467-2009 influenza A(H1N1)-M	(38)	TCTCGTCATTGCAGCAAATATCATTGGGATCTTGCACCTG
301-6469-2009 influenza A(H1N1)-M	(38)	TCTCGTCATTGCAGCAAATATCATTGGGATCTTGCACCTG
301-6509-2009 influenza A(H1N1)-M	(38)	TCTCGTCATTGCAGCAAATATCATTGGGATCTTGCACCTG
301-6519-2009 influenza A(H1N1)-M	(39)	TCTCGTCATTGCAGCAAATATCATTGGGATCTTGCACCTG
301-6525-2009 influenza A(H1N1)-M	(38)	TCTCGTCATTGCAGCAAATATCATTGGGATCTTGCACCTG
301-6527-2009 influenza A(H1N1)-M	(37)	TCTCGTCATTGCAGCAAATATCATTGGGATCTTGCACCTG
301-6532-2009 influenza A(H1N1)-M	(38)	TCTCGTCATTGCAGCAAATATCATTGGGATCTTGCACCTG

27V 31N

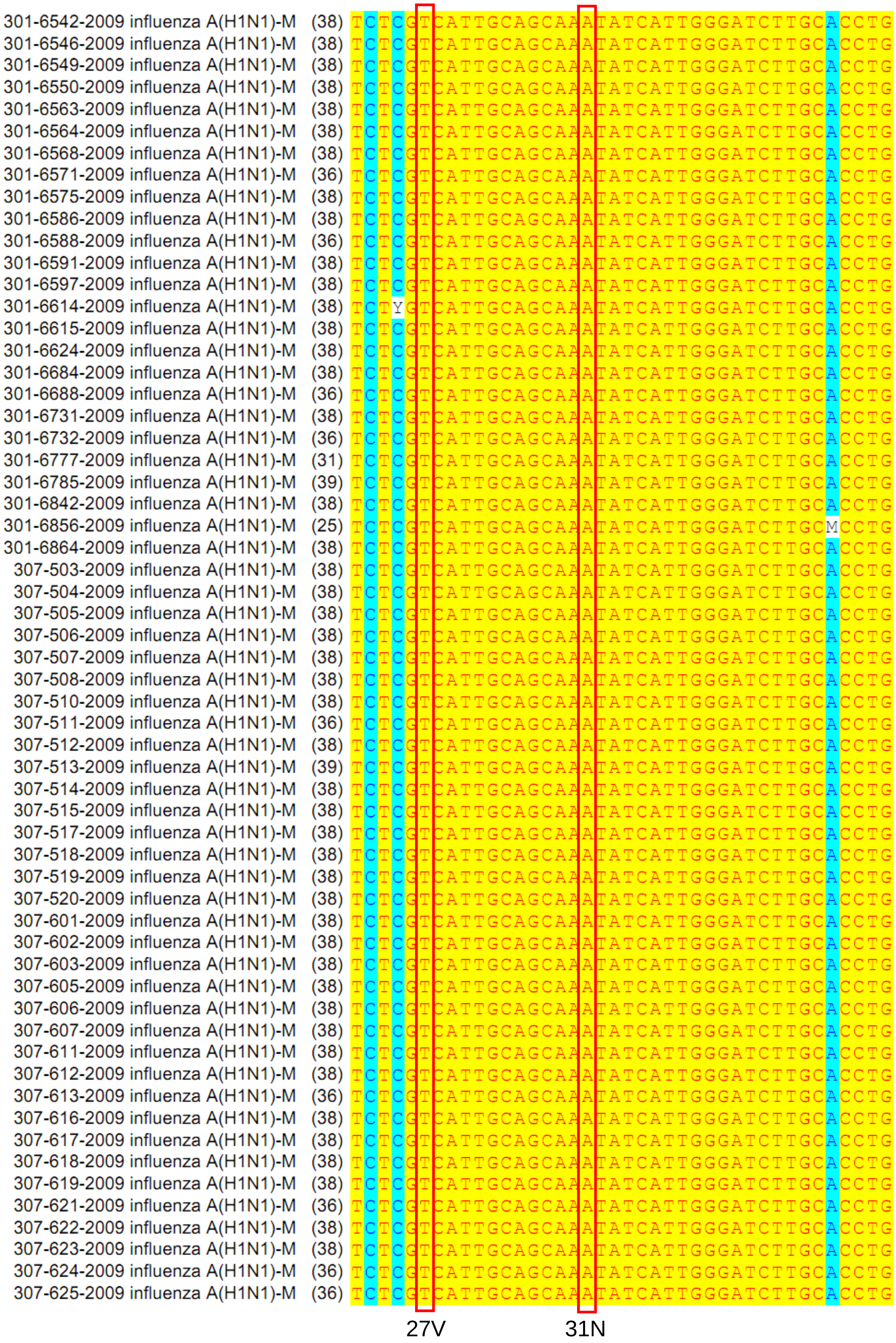

301-6542-2009 influenza A(H1N1)-M (38) TCTCGTCATTGCAGCAAATATCATTGGGATCTTGCACCTG
301-6546-2009 influenza A(H1N1)-M (38) TCTCGTCATTGCAGCAAATATCATTGGGATCTTGCACCTG
301-6549-2009 influenza A(H1N1)-M (38) TCTCGTCATTGCAGCAAATATCATTGGGATCTTGCACCTG
301-6550-2009 influenza A(H1N1)-M (38) TCTCGTCATTGCAGCAAATATCATTGGGATCTTGCACCTG
301-6563-2009 influenza A(H1N1)-M (38) TCTCGTCATTGCAGCAAATATCATTGGGATCTTGCACCTG
301-6564-2009 influenza A(H1N1)-M (38) TCTCGTCATTGCAGCAAATATCATTGGGATCTTGCACCTG
301-6568-2009 influenza A(H1N1)-M (38) TCTCGTCATTGCAGCAAATATCATTGGGATCTTGCACCTG
301-6571-2009 influenza A(H1N1)-M (36) TCTCGTCATTGCAGCAAATATCATTGGGATCTTGCACCTG
301-6575-2009 influenza A(H1N1)-M (38) TCTCGTCATTGCAGCAAATATCATTGGGATCTTGCACCTG
301-6586-2009 influenza A(H1N1)-M (38) TCTCGTCATTGCAGCAAATATCATTGGGATCTTGCACCTG
301-6588-2009 influenza A(H1N1)-M (36) TCTCGTCATTGCAGCAAATATCATTGGGATCTTGCACCTG
301-6591-2009 influenza A(H1N1)-M (38) TCTCGTCATTGCAGCAAATATCATTGGGATCTTGCACCTG
301-6597-2009 influenza A(H1N1)-M (38) TCTCGTCATTGCAGCAAATATCATTGGGATCTTGCACCTG
301-6614-2009 influenza A(H1N1)-M (38) TCTYGTCATTGCAGCAAATATCATTGGGATCTTGCACCTG
301-6615-2009 influenza A(H1N1)-M (38) TCTCGTCATTGCAGCAAATATCATTGGGATCTTGCACCTG
301-6624-2009 influenza A(H1N1)-M (38) TCTCGTCATTGCAGCAAATATCATTGGGATCTTGCACCTG
301-6684-2009 influenza A(H1N1)-M (38) TCTCGTCATTGCAGCAAATATCATTGGGATCTTGCACCTG
301-6688-2009 influenza A(H1N1)-M (36) TCTCGTCATTGCAGCAAATATCATTGGGATCTTGCACCTG
301-6731-2009 influenza A(H1N1)-M (38) TCTCGTCATTGCAGCAAATATCATTGGGATCTTGCACCTG
301-6732-2009 influenza A(H1N1)-M (36) TCTCGTCATTGCAGCAAATATCATTGGGATCTTGCACCTG
301-6777-2009 influenza A(H1N1)-M (31) TCTCGTCATTGCAGCAAATATCATTGGGATCTTGCACCTG
301-6785-2009 influenza A(H1N1)-M (39) TCTCGTCATTGCAGCAAATATCATTGGGATCTTGCACCTG
301-6842-2009 influenza A(H1N1)-M (38) TCTCGTCATTGCAGCAAATATCATTGGGATCTTGCACCTG
301-6856-2009 influenza A(H1N1)-M (25) TCTCGTCATTGCAGCAAATATCATTGGGATCTTGCMCCTG
301-6864-2009 influenza A(H1N1)-M (38) TCTCGTCATTGCAGCAAATATCATTGGGATCTTGCACCTG
307-503-2009 influenza A(H1N1)-M (38) TCTCGTCATTGCAGCAAATATCATTGGGATCTTGCACCTG
307-504-2009 influenza A(H1N1)-M (38) TCTCGTCATTGCAGCAAATATCATTGGGATCTTGCACCTG
307-505-2009 influenza A(H1N1)-M (38) TCTCGTCATTGCAGCAAATATCATTGGGATCTTGCACCTG
307-506-2009 influenza A(H1N1)-M (38) TCTCGTCATTGCAGCAAATATCATTGGGATCTTGCACCTG
307-507-2009 influenza A(H1N1)-M (38) TCTCGTCATTGCAGCAAATATCATTGGGATCTTGCACCTG
307-508-2009 influenza A(H1N1)-M (38) TCTCGTCATTGCAGCAAATATCATTGGGATCTTGCACCTG
307-510-2009 influenza A(H1N1)-M (38) TCTCGTCATTGCAGCAAATATCATTGGGATCTTGCACCTG
307-511-2009 influenza A(H1N1)-M (36) TCTCGTCATTGCAGCAAATATCATTGGGATCTTGCACCTG
307-512-2009 influenza A(H1N1)-M (38) TCTCGTCATTGCAGCAAATATCATTGGGATCTTGCACCTG
307-513-2009 influenza A(H1N1)-M (39) TCTCGTCATTGCAGCAAATATCATTGGGATCTTGCACCTG
307-514-2009 influenza A(H1N1)-M (38) TCTCGTCATTGCAGCAAATATCATTGGGATCTTGCACCTG
307-515-2009 influenza A(H1N1)-M (38) TCTCGTCATTGCAGCAAATATCATTGGGATCTTGCACCTG
307-517-2009 influenza A(H1N1)-M (38) TCTCGTCATTGCAGCAAATATCATTGGGATCTTGCACCTG
307-518-2009 influenza A(H1N1)-M (38) TCTCGTCATTGCAGCAAATATCATTGGGATCTTGCACCTG
307-519-2009 influenza A(H1N1)-M (38) TCTCGTCATTGCAGCAAATATCATTGGGATCTTGCACCTG
307-520-2009 influenza A(H1N1)-M (38) TCTCGTCATTGCAGCAAATATCATTGGGATCTTGCACCTG
307-601-2009 influenza A(H1N1)-M (38) TCTCGTCATTGCAGCAAATATCATTGGGATCTTGCACCTG
307-602-2009 influenza A(H1N1)-M (38) TCTCGTCATTGCAGCAAATATCATTGGGATCTTGCACCTG
307-603-2009 influenza A(H1N1)-M (38) TCTCGTCATTGCAGCAAATATCATTGGGATCTTGCACCTG
307-605-2009 influenza A(H1N1)-M (38) TCTCGTCATTGCAGCAAATATCATTGGGATCTTGCACCTG
307-606-2009 influenza A(H1N1)-M (38) TCTCGTCATTGCAGCAAATATCATTGGGATCTTGCACCTG
307-607-2009 influenza A(H1N1)-M (38) TCTCGTCATTGCAGCAAATATCATTGGGATCTTGCACCTG
307-611-2009 influenza A(H1N1)-M (38) TCTCGTCATTGCAGCAAATATCATTGGGATCTTGCACCTG
307-612-2009 influenza A(H1N1)-M (38) TCTCGTCATTGCAGCAAATATCATTGGGATCTTGCACCTG
307-613-2009 influenza A(H1N1)-M (36) TCTCGTCATTGCAGCAAATATCATTGGGATCTTGCACCTG
307-616-2009 influenza A(H1N1)-M (38) TCTCGTCATTGCAGCAAATATCATTGGGATCTTGCACCTG
307-617-2009 influenza A(H1N1)-M (38) TCTCGTCATTGCAGCAAATATCATTGGGATCTTGCACCTG
307-618-2009 influenza A(H1N1)-M (38) TCTCGTCATTGCAGCAAATATCATTGGGATCTTGCACCTG
307-619-2009 influenza A(H1N1)-M (38) TCTCGTCATTGCAGCAAATATCATTGGGATCTTGCACCTG
307-621-2009 influenza A(H1N1)-M (36) TCTCGTCATTGCAGCAAATATCATTGGGATCTTGCACCTG
307-622-2009 influenza A(H1N1)-M (38) TCTCGTCATTGCAGCAAATATCATTGGGATCTTGCACCTG
307-623-2009 influenza A(H1N1)-M (38) TCTCGTCATTGCAGCAAATATCATTGGGATCTTGCACCTG
307-624-2009 influenza A(H1N1)-M (36) TCTCGTCATTGCAGCAAATATCATTGGGATCTTGCACCTG
307-625-2009 influenza A(H1N1)-M (36) TCTCGTCATTGCAGCAAATATCATTGGGATCTTGCACCTG
27V
31N

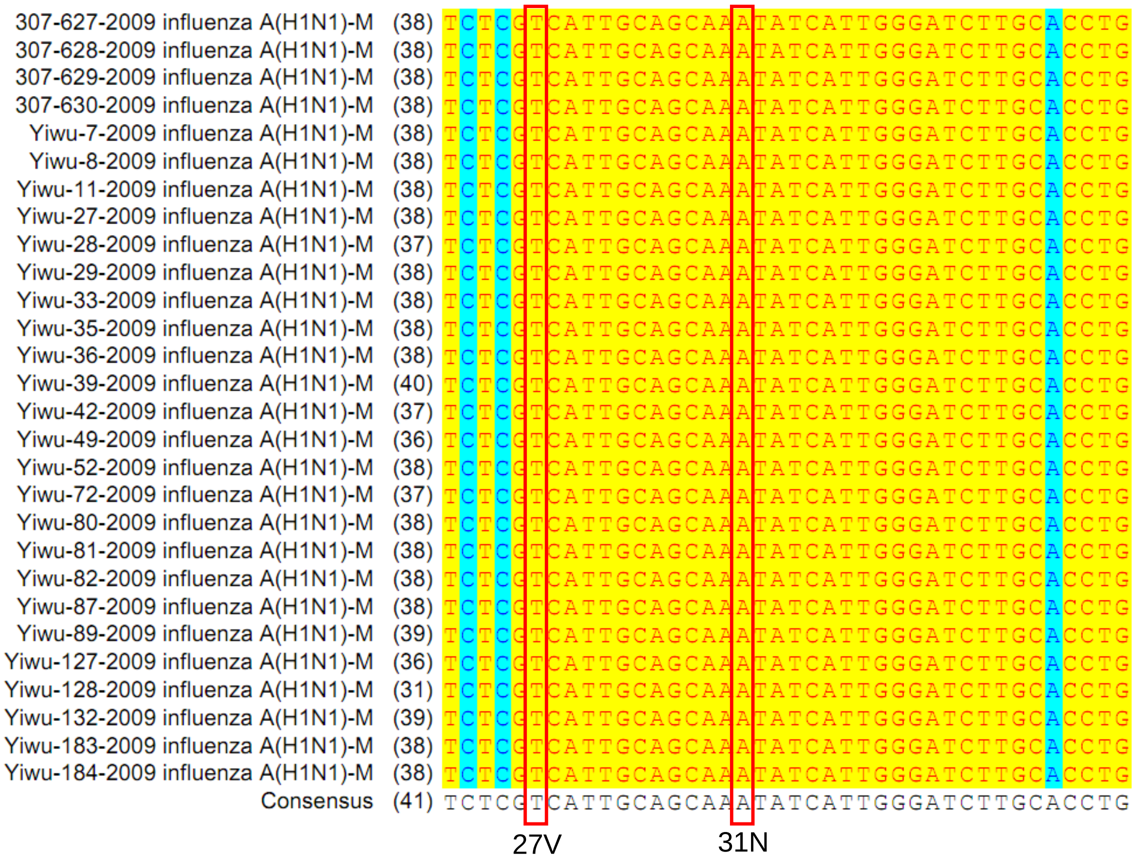
307-627-2009 influenza A(H1N1)-M (38) TCTCGTCATTGCAGCAAATATCATTGGGATCTTGCACCTG
307-628-2009 influenza A(H1N1)-M (38) TCTCGTCATTGCAGCAAATATCATTGGGATCTTGCACCTG
307-629-2009 influenza A(H1N1)-M (38) TCTCGTCATTGCAGCAAATATCATTGGGATCTTGCACCTG
307-630-2009 influenza A(H1N1)-M (38) TCTCGTCATTGCAGCAAATATCATTGGGATCTTGCACCTG
Yiwu-7-2009 influenza A(H1N1)-M (38) TCTCGTCATTGCAGCAAATATCATTGGGATCTTGCACCTG
Yiwu-8-2009 influenza A(H1N1)-M (38) TCTCGTCATTGCAGCAAATATCATTGGGATCTTGCACCTG
Yiwu-11-2009 influenza A(H1N1)-M (38) TCTCGTCATTGCAGCAAATATCATTGGGATCTTGCACCTG
Yiwu-27-2009 influenza A(H1N1)-M (38) TCTCGTCATTGCAGCAAATATCATTGGGATCTTGCACCTG
Yiwu-28-2009 influenza A(H1N1)-M (37) TCTCGTCATTGCAGCAAATATCATTGGGATCTTGCACCTG
Yiwu-29-2009 influenza A(H1N1)-M (38) TCTCGTCATTGCAGCAAATATCATTGGGATCTTGCACCTG
Yiwu-33-2009 influenza A(H1N1)-M (38) TCTCGTCATTGCAGCAAATATCATTGGGATCTTGCACCTG
Yiwu-35-2009 influenza A(H1N1)-M (38) TCTCGTCATTGCAGCAAATATCATTGGGATCTTGCACCTG
Yiwu-36-2009 influenza A(H1N1)-M (38) TCTCGTCATTGCAGCAAATATCATTGGGATCTTGCACCTG
Yiwu-39-2009 influenza A(H1N1)-M (40) TCTCGTCATTGCAGCAAATATCATTGGGATCTTGCACCTG
Yiwu-42-2009 influenza A(H1N1)-M (37) TCTCGTCATTGCAGCAAATATCATTGGGATCTTGCACCTG
Yiwu-49-2009 influenza A(H1N1)-M (36) TCTCGTCATTGCAGCAAATATCATTGGGATCTTGCACCTG
Yiwu-52-2009 influenza A(H1N1)-M (38) TCTCGTCATTGCAGCAAATATCATTGGGATCTTGCACCTG
Yiwu-72-2009 influenza A(H1N1)-M (37) TCTCGTCATTGCAGCAAATATCATTGGGATCTTGCACCTG
Yiwu-80-2009 influenza A(H1N1)-M (38) TCTCGTCATTGCAGCAAATATCATTGGGATCTTGCACCTG
Yiwu-81-2009 influenza A(H1N1)-M (38) TCTCGTCATTGCAGCAAATATCATTGGGATCTTGCACCTG
Yiwu-82-2009 influenza A(H1N1)-M (38) TCTCGTCATTGCAGCAAATATCATTGGGATCTTGCACCTG
Yiwu-87-2009 influenza A(H1N1)-M (38) TCTCGTCATTGCAGCAAATATCATTGGGATCTTGCACCTG
Yiwu-89-2009 influenza A(H1N1)-M (39) TCTCGTCATTGCAGCAAATATCATTGGGATCTTGCACCTG
Yiwu-127-2009 influenza A(H1N1)-M (36) TCTCGTCATTGCAGCAAATATCATTGGGATCTTGCACCTG
Yiwu-128-2009 influenza A(H1N1)-M (31) TCTCGTCATTGCAGCAAATATCATTGGGATCTTGCACCTG
Yiwu-132-2009 influenza A(H1N1)-M (39) TCTCGTCATTGCAGCAAATATCATTGGGATCTTGCACCTG
Yiwu-183-2009 influenza A(H1N1)-M (38) TCTCGTCATTGCAGCAAATATCATTGGGATCTTGCACCTG
Yiwu-184-2009 influenza A(H1N1)-M (38) TCTCGTCATTGCAGCAAATATCATTGGGATCTTGCACCTG
Consensus (41) TCTCGTCATTGCAGCAAATATCATTGGGATCTTGCACCTG
27V
31N

2009 influenza A (H1N1) NA gene

	(118)	118 130 140 156
301-75-2009 influenza A(H1N1)-NA	(97)	ATGCCCCTAATTATCACTATGAGGAATGCTCCTGTTATC
301-2654-2009 influenza A(H1N1)-NA	(99)	ATGCCCCTAATTATCACTATGAGGAATGCTCCTGTTATC
301-2655-2009 influenza A(H1N1)-NA	(99)	ATGCCCCTAATTATCACTATGAGGAATGCTCCTGTTATC
301-2747-2009 influenza A(H1N1)-NA	(96)	ATGCCCCTAATTATCACTATGAGGAATGCTCCTGTTATC
301-2749-2009 influenza A(H1N1)-NA	(98)	ATGCCCCTAATTATCACTATGAGGAATGCTCCTGTTATC
301-2750-2009 influenza A(H1N1)-NA	(101)	ATGCCCCTAATTATCACTATGAGGAATGCTCCTGTTATC
301-2751-2009 influenza A(H1N1)-NA	(97)	-TGCCCCTAATTATCACTATGAGGA-TGCTCCTGTTATC
301-2760-2009 influenza A(H1N1)-NA	(105)	ATGCCCCTAATTATCACTATGAGGAATGCTCCTGTTATC
301-2767-2009 influenza A(H1N1)-NA	(97)	ATGCCCCTAATTATCACTATGAGGAATGCTCCTGTTATC
301-2770-2009 influenza A(H1N1)-NA	(100)	ATGCCCCTAATTATCACTATGAGGAATGCTCCTGTTATC
301-2777-2009 influenza A(H1N1)-NA	(102)	ATGCCCCTAATTATCACTATGAGGAATGCTCCTGTTATC
301-2796-2009 influenza A(H1N1)-NA	(98)	ATGCCCCTAATTATCACTATGAGGAATGCTCCTGTTATC
301-2807-2009 influenza A(H1N1)-NA	(101)	ATGCCCCTAATTATCACTATGAGGAATGCTCCTGTTATC
301-2833-2009 influenza A(H1N1)-NA	(102)	ATGCCCCTAATTATCACTATGAGGAATGCTCCTGTTATC
301-2835-2009 influenza A(H1N1)-NA	(96)	ATGCCCCTAATTATCACTATGAGGAATGCTCCTGTTATC
301-2836-2009 influenza A(H1N1)-NA	(99)	ATGCCCCTAATTATCACTATGAGGAATGCTCCTGTTATC
301-2837-2009 influenza A(H1N1)-NA	(101)	ATGCCCCTAATTATCACTATGAGGAATGCTCCTGTTATC
301-2838-2009 influenza A(H1N1)-NA	(104)	ATGCCCCTAATTATCACTATGAGGAATGCTCCTGTTATC
301-2840-2009 influenza A(H1N1)-NA	(99)	ATGCCCCTAATTATCACTATGAGGAATGCTCCTGTTATC
301-2856-2009 influenza A(H1N1)-NA	(102)	ATGCCCCTAATTATCACTATGAGGAATGCTCCTGTTATC
301-2858-2009 influenza A(H1N1)-NA	(98)	ATGCCCCTAATTATCACTATGAGGAATGCTCCTGTTATC
301-2860-2009 influenza A(H1N1)-NA	(102)	ATGCCCCTAATTATCACTATGAGGAATGCTCCTGTTATC
301-2867-2009 influenza A(H1N1)-NA	(101)	ATGCCCCTAATTATCACTATGAGGAATGCTCCTGTTATC
301-2884-2009 influenza A(H1N1)-NA	(101)	ATGCCCCTAATTATCACTATGAGGAATGCTCCTGTTATC
301-2886-2009 influenza A(H1N1)-NA	(101)	ATGCCCCTAATTATCACTATGAGGAATGCTCCTGTTATC
301-2887-2009 influenza A(H1N1)-NA	(98)	-TGCCCCTAATTATCACTATGAGGAATGCTCCTGTTATC
301-2930-2009 influenza A(H1N1)-NA	(103)	ATGCCCCTAATTATCACTATGAGGAATGCTCCTGTTATC
301-2931-2009 influenza A(H1N1)-NA	(101)	ATGCCCCTAATTATCACTATGAGGAATGCTCCTGTTATC
301-2932-2009 influenza A(H1N1)-NA	(101)	ATGCCCCTAATTATCACTATGAGGAATGCTCCTGTTATC
301-3291-2009 influenza A(H1N1)-NA	(97)	ATGCCCCTAATTATCACTATGAGGAATGCTCCTGTTATC
301-3294-2009 influenza A(H1N1)-NA	(100)	ATGCCCCTAATTATCACTATGAGGAATGCTCCTGTTATC
301-3310-2009 influenza A(H1N1)-NA	(101)	ATGCCCCTAATTATCACTATGAGGAATGCTCCTGTTATC
301-3320-2009 influenza A(H1N1)-NA	(102)	ATGCCCCTAATTATCACTATGAGGAATGCTCCTGTTATC
301-3322-2009 influenza A(H1N1)-NA	(97)	ATGCCCCTAATTATCACTATGAGGAATGCTCCTGTTATC
301-3324-2009 influenza A(H1N1)-NA	(101)	ATGCCCCTAATTATCACTATGAGGAATGCTCCTGTTATC
301-3327-2009 influenza A(H1N1)-NA	(102)	ATGCCCCTAATTATCACTATGAGGAATGCTCCTGTTATC
301-3332-2009 influenza A(H1N1)-NA	(99)	ATGCCCCTAATTATCACTATGAGGAATGCTCCTGTTATC
301-3336-2009 influenza A(H1N1)-NA	(99)	ATGCCCCTAATTATCACTATGAGGAATGCTCCTGTTATC
301-3337-2009 influenza A(H1N1)-NA	(100)	ATGCCCCTAATTATCACTATGAGGAATGCTCCTGTTATC
301-6078-2009 influenza A(H1N1)-NA	(101)	ATGCCCCTAATTATCACTATGAGGAATGCTCCTGTTATC
301-6202-2009 influenza A(H1N1)-NA	(99)	ATGCCCCTAATTATCACTATGAGGAATGCTCCTGTTATC
301-6223-2009 influenza A(H1N1)-NA	(101)	ATGCCCCTAATTATCACTATGAGGAATGCTCCTGTTATC
301-6228-2009 influenza A(H1N1)-NA	(104)	ATGCCCCTAATTATCACTATGAGGAATGCTCCTGTTATC
301-6241-2009 influenza A(H1N1)-NA	(102)	ATGCCCCTAATTATCACTATGAGGAATGCTCCTGCTATC
301-6259-2009 influenza A(H1N1)-NA	(97)	ATGCCCCTAATTATCACTATGAGGAATGCTCCTGTTATC
301-6284-2009 influenza A(H1N1)-NA	(98)	ATGCCCCTAATTATCACTATGAGGAATGCTCCTGTTATC
301-6298-2009 influenza A(H1N1)-NA	(100)	ATGCCCCTAATTATCACTATGAGGAATGCTCCTGTTATC
301-6319-2009 influenza A(H1N1)-NA	(103)	ATGCCCCTAATTATCACTATGAGGAATGCTCCTGCTATC
301-6336-2009 influenza A(H1N1)-NA	(103)	ATGCCCCTAATTATCACTATGAGGAATGCTCCTGTTATC
301-6379-2009 influenza A(H1N1)-NA	(101)	ATGCCCCTAATTATCACTATGAGGAATGCTCCTGTTATC
301-6467-2009 influenza A(H1N1)-NA	(97)	ATGCCCCTAATTATCACTATGAGGAATGCTCCTGTTATC
301-6469-2009 influenza A(H1N1)-NA	(99)	ATGCCCCTAATTATCACTATGAGGAATGCTCCTGTTATC
301-6509-2009 influenza A(H1N1)-NA	(95)	ATGCCCCTAATTATCACTATGAGGAATGCTCCTGTTATC
301-6519-2009 influenza A(H1N1)-NA	(102)	ATGCCCCTAATTATCACTATGAGGAATGCTCCTGTTATC
301-6525-2009 influenza A(H1N1)-NA	(95)	ATGCCCCTAATTATCACTATGAGGAATGCTCCTGTTATC
301-6527-2009 influenza A(H1N1)-NA	(99)	ATGCCCCTAATTATCACTATGAGGAATGCTCCTGTTATC
301-6532-2009 influenza A(H1N1)-NA	(102)	ATGCCCCTAATTATCACTATGAGGAATGCTCCTGTTATC

274H

301-6542-2009 influenza A(H1N1)-NA (104)	ATGCCCCTAATTATCACTATGAGGAATGCTCCTGTTATC
301-6546-2009 influenza A(H1N1)-NA (95)	ATGCCCCTAATTATCACTATGAGGAATGCTCCTGCTATC
301-6549-2009 influenza A(H1N1)-NA (98)	ATGCCCCTAATTATCACTATGAGGAATGCTCCTGTTATC
301-6550-2009 influenza A(H1N1)-NA (102)	ATGCCCCTAATTATCACTATGAGGAATGCTCCTGTTATC
301-6563-2009 influenza A(H1N1)-NA (104)	ATGCCCCTAATTATCACTATGAGGAATGCTCCTGTTATC
301-6564-2009 influenza A(H1N1)-NA (97)	ATGCCCCTAATTATCACTATGAGGAATGCTCCTGTTATC
301-6568-2009 influenza A(H1N1)-NA (103)	ATGCCCCTAATTATCACTATGAGGAATGCTCCTGTTATC
301-6571-2009 influenza A(H1N1)-NA (101)	ATGCCCCTAATTATCACTATGAGGAATGCTCCTGTTATC
301-6575-2009 influenza A(H1N1)-NA (101)	ATGCCCCTAATTATCACTATGAGGAATGCTCCTGTTATC
301-6586-2009 influenza A(H1N1)-NA (102)	ATGCCCCTAATTATCACTATGAGGAATGCTCCTGTTATC
301-6588-2009 influenza A(H1N1)-NA (100)	ATGCCCCTAATTATCACTATGAGGAATGCTCCTGTTATC
301-6591-2009 influenza A(H1N1)-NA (100)	ATGCCCCTAATTATCACTATGAGGAATGCTCCTGTTATC
301-6597-2009 influenza A(H1N1)-NA (99)	ATGCCCCTAATTATCACTATGAGGAATGCTCCTGTTATC
301-6614-2009 influenza A(H1N1)-NA (101)	ATGCCCCTAATTATCACTATGAGGAATGCTCCTGTTATC
301-6615-2009 influenza A(H1N1)-NA (101)	ATGCCCCTAATTATCACTATGAGGAATGCTCCTGTTATC
301-6624-2009 influenza A(H1N1)-NA (101)	ATGCCCCTAATTATCACTATGAGGAATGCTCCTGTTATC
301-6684-2009 influenza A(H1N1)-NA (107)	ATGCCCCTAATTATCACTATGAGGAATGCTCCTGTTATC
301-6688-2009 influenza A(H1N1)-NA (102)	ATGCCCCTAATTATCACTATGAGGAATGCTCCTGTTATC
301-6731-2009 influenza A(H1N1)-NA (100)	ATGCCCCTAATTATCACTATGAGGAATGCTCCTGTTATC
301-6732-2009 influenza A(H1N1)-NA (99)	ATGCCCCTAATTATCACTATGAGGAATGCTCCTGTTATC
301-6777-2009 influenza A(H1N1)-NA (99)	ATGCCCCTAATTATCACTATGAGGAATGCTCCTGTTATC
301-6785-2009 influenza A(H1N1)-NA (100)	ATGCCCCTAATTATCACTATGAGGAATGCTCCTGTTATC
301-6842-2009 influenza A(H1N1)-NA (102)	ATGCCCCTAATTATCACTATGAGGAATGCTCCTGTTATC
301-6856-2009 influenza A(H1N1)-NA (102)	ATGCCCCTAATTATCACTATGAGGAATGCTCCTGTTATC
301-6864-2009 influenza A(H1N1)-NA (102)	ATGCCCCTAATTATCACTATGAGGAATGCTCCTGTTATC
307-503-2009 influenza A(H1N1)-NA (99)	ATGCCCCTAATTATCACTATGAGGAATGCTCCTGTTATC
307-504-2009 influenza A(H1N1)-NA (101)	ATGCCCCTAATTATCACTATGAGGAATGCTCCTGTTATC
307-505-2009 influenza A(H1N1)-NA (98)	ATGCCCCTAATTATCACTATGAGGAATGCTCCTGTTATC
307-506-2009 influenza A(H1N1)-NA (99)	ATGCCCCTAATTATCACTATGAGGAATGCTCCTGTTATC
307-507-2009 influenza A(H1N1)-NA (101)	ATGCCCCTAATTATCACTATGAGGAATGCTCCTGTTATC
307-508-2009 influenza A(H1N1)-NA (97)	ATGCCCCTAATTATCACTATGAGGAATGCTCCTGTTATC
307-510-2009 influenza A(H1N1)-NA (99)	ATGCCCCTAATTATCACTATGAGGAATGCTCCTGTTATC
307-511-2009 influenza A(H1N1)-NA (98)	ATGCCCCTAATTATCACTATGAGGAATGCTCCTGTTATC
307-512-2009 influenza A(H1N1)-NA (102)	ATGCCCCTAATTATCACTATGAGGAATGCTCCTGCTATC
307-513-2009 influenza A(H1N1)-NA (102)	ATGCCCCTAATTATCACTATGAGGAATGCTCCTGTTATC
307-514-2009 influenza A(H1N1)-NA (101)	ATGCCCCTAATTATCACTATGAGGAATGCTCCTGTTATC
307-515-2009 influenza A(H1N1)-NA (96)	ATGCCCCTAATTATCACTATGAGGAATGCTCCTGTTATC
307-517-2009 influenza A(H1N1)-NA (102)	ATGCCCCTAATTATCACTATGAGGAATGCTCCTGTTATC
307-518-2009 influenza A(H1N1)-NA (101)	ATGCCCCTAATTATCACTATGAGGAATGCTCCTGTTATC
307-519-2009 influenza A(H1N1)-NA (100)	ATGCCCCTAATTATCACTATGAGGAATGCTCCTGTTATC
307-520-2009 influenza A(H1N1)-NA (100)	ATGCCCCTAATTATCACTATGAGGAATGCTCCTGTTATC
307-601-2009 influenza A(H1N1)-NA (101)	ATGCCCCTAATTATCACTATGAGGAATGCTCCTGTTATC
307-602-2009 influenza A(H1N1)-NA (97)	ATGCCCCTAATTATCACTATGAGGAATGCTCCTGTTATC
307-603-2009 influenza A(H1N1)-NA (98)	ATGCCCCTAATTATCACTATGAGGAATGCTCCTGTTATC
307-605-2009 influenza A(H1N1)-NA (103)	ATGCCCCTAATTATCACTATGAGGAATGCTCCTGTTATC
307-606-2009 influenza A(H1N1)-NA (98)	ATGCCCCTAATTATCACTATGAGGAATGCTCCTGTTATC
307-607-2009 influenza A(H1N1)-NA (100)	ATGCCCCTAATTATCACTATGAGGAATGCTCCTGTTATC
307-611-2009 influenza A(H1N1)-NA (99)	ATGCCCCTAATTATCACTATGAGGAATGCTCCTGTTATC
307-612-2009 influenza A(H1N1)-NA (101)	ATGCCCCTAATTATCACTATGAGGAATGCTCCTGTTATC
307-613-2009 influenza A(H1N1)-NA (102)	ATGCCCCTAATTATCACTATGAGGAATGCTCCTGTTATC
307-616-2009 influenza A(H1N1)-NA (101)	ATGCCCCTAATTATCACTATGAGGAATGCTCCTGTTATC
307-617-2009 influenza A(H1N1)-NA (99)	ATGCCCCTAATTATCACTATGAGGAATGCTCCTGTTATC
307-618-2009 influenza A(H1N1)-NA (101)	ATGCCCCTAATTATCACTATGAGGAATGCTCCTGTTATC
307-619-2009 influenza A(H1N1)-NA (102)	ATGCCCCTAATTATCACTATGAGGAATGCTCCTGTTATC
307-621-2009 influenza A(H1N1)-NA (101)	ATGCCCCTAATTATCACTATGAGGAATGCTCCTGTTATC
307-622-2009 influenza A(H1N1)-NA (98)	ATGCCCCTAATTATCACTATGAGGAATGCTCCTGTTATC
307-623-2009 influenza A(H1N1)-NA (102)	ATGCCCCTAATTATCACTATGAGGAATGCTCCTGTTATC
307-624-2009 influenza A(H1N1)-NA (105)	ATGCCCCTAATTATCACTATGAGGAATGCTCCTGTTATC
307-625-2009 influenza A(H1N1)-NA (97)	ATGCCCCTAATTATCACTATGAGGAATGCTCCTGTTATC

274H

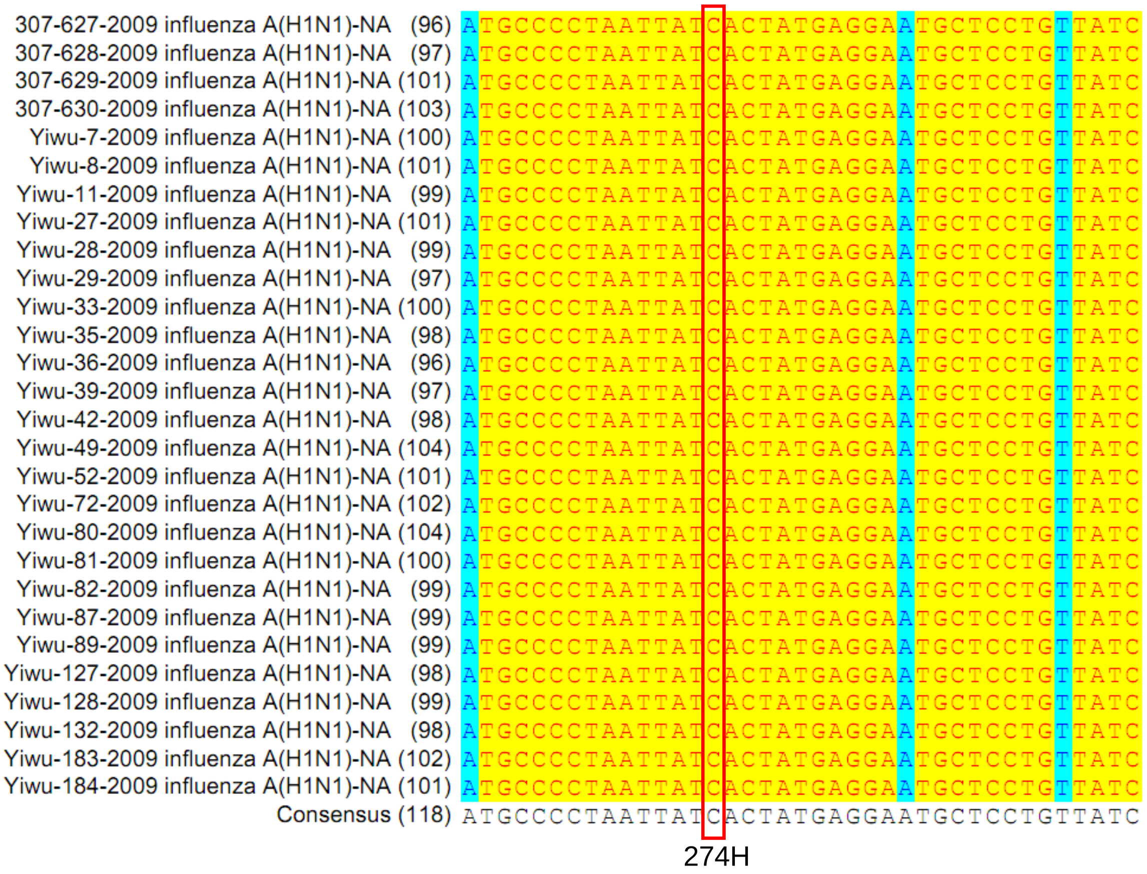

307-627-2009 influenza A(H1N1)-NA (96) ATGCCCCTAATTATCACTATGAGGAATGCTCCTGTTATC
307-628-2009 influenza A(H1N1)-NA (97) ATGCCCCTAATTATCACTATGAGGAATGCTCCTGTTATC
307-629-2009 influenza A(H1N1)-NA (101) ATGCCCCTAATTATCACTATGAGGAATGCTCCTGTTATC
307-630-2009 influenza A(H1N1)-NA (103) ATGCCCCTAATTATCACTATGAGGAATGCTCCTGTTATC
Yiwu-7-2009 influenza A(H1N1)-NA (100) ATGCCCCTAATTATCACTATGAGGAATGCTCCTGTTATC
Yiwu-8-2009 influenza A(H1N1)-NA (101) ATGCCCCTAATTATCACTATGAGGAATGCTCCTGTTATC
Yiwu-11-2009 influenza A(H1N1)-NA (99) ATGCCCCTAATTATCACTATGAGGAATGCTCCTGTTATC
Yiwu-27-2009 influenza A(H1N1)-NA (101) ATGCCCCTAATTATCACTATGAGGAATGCTCCTGTTATC
Yiwu-28-2009 influenza A(H1N1)-NA (99) ATGCCCCTAATTATCACTATGAGGAATGCTCCTGTTATC
Yiwu-29-2009 influenza A(H1N1)-NA (97) ATGCCCCTAATTATCACTATGAGGAATGCTCCTGTTATC
Yiwu-33-2009 influenza A(H1N1)-NA (100) ATGCCCCTAATTATCACTATGAGGAATGCTCCTGTTATC
Yiwu-35-2009 influenza A(H1N1)-NA (98) ATGCCCCTAATTATCACTATGAGGAATGCTCCTGTTATC
Yiwu-36-2009 influenza A(H1N1)-NA (96) ATGCCCCTAATTATCACTATGAGGAATGCTCCTGTTATC
Yiwu-39-2009 influenza A(H1N1)-NA (97) ATGCCCCTAATTATCACTATGAGGAATGCTCCTGTTATC
Yiwu-42-2009 influenza A(H1N1)-NA (98) ATGCCCCTAATTATCACTATGAGGAATGCTCCTGTTATC
Yiwu-49-2009 influenza A(H1N1)-NA (104) ATGCCCCTAATTATCACTATGAGGAATGCTCCTGTTATC
Yiwu-52-2009 influenza A(H1N1)-NA (101) ATGCCCCTAATTATCACTATGAGGAATGCTCCTGTTATC
Yiwu-72-2009 influenza A(H1N1)-NA (102) ATGCCCCTAATTATCACTATGAGGAATGCTCCTGTTATC
Yiwu-80-2009 influenza A(H1N1)-NA (104) ATGCCCCTAATTATCACTATGAGGAATGCTCCTGTTATC
Yiwu-81-2009 influenza A(H1N1)-NA (100) ATGCCCCTAATTATCACTATGAGGAATGCTCCTGTTATC
Yiwu-82-2009 influenza A(H1N1)-NA (99) ATGCCCCTAATTATCACTATGAGGAATGCTCCTGTTATC
Yiwu-87-2009 influenza A(H1N1)-NA (99) ATGCCCCTAATTATCACTATGAGGAATGCTCCTGTTATC
Yiwu-89-2009 influenza A(H1N1)-NA (99) ATGCCCCTAATTATCACTATGAGGAATGCTCCTGTTATC
Yiwu-127-2009 influenza A(H1N1)-NA (98) ATGCCCCTAATTATCACTATGAGGAATGCTCCTGTTATC
Yiwu-128-2009 influenza A(H1N1)-NA (99) ATGCCCCTAATTATCACTATGAGGAATGCTCCTGTTATC
Yiwu-132-2009 influenza A(H1N1)-NA (98) ATGCCCCTAATTATCACTATGAGGAATGCTCCTGTTATC
Yiwu-183-2009 influenza A(H1N1)-NA (102) ATGCCCCTAATTATCACTATGAGGAATGCTCCTGTTATC
Yiwu-184-2009 influenza A(H1N1)-NA (101) ATGCCCCTAATTATCACTATGAGGAATGCTCCTGTTATC
Consensus (118) ATGCCCCTAATTATCACTATGAGGAATGCTCCTGTTATC
274H
